# Potential Benefits of Bovine Colostrum in Pediatric Nutrition and Health

**DOI:** 10.3390/nu13082551

**Published:** 2021-07-26

**Authors:** Per Torp Sangild, Caitlin Vonderohe, Valeria Melendez Hebib, Douglas G. Burrin

**Affiliations:** 1 Comparative Pediatrics & Nutrition, University of Copenhagen, DK-1870 Copenhagen, Denmark; pts@sund.ku.dk; 2 Department of Neonatology, Rigshospitalet, DK-1870 Copenhagen, Denmark; 3 Department of Pediatrics, Odense University Hospital, DK-5000 Odense, Denmark; 4 USDA-ARS Children’s Nutrition Research Center, Pediatrics, Gastroenterology & Nutrition, Baylor College of Medicine, Houston, TX 77030, USA; Caitlin.Vonderohe@bcm.edu (C.V.); Valeria.Melendez@bcm.edu (V.M.H.)

**Keywords:** preterm infants, human milk, immunoglobulins, necrotizing enterocolitis, diarrhea

## Abstract

Bovine colostrum (BC), the first milk produced from cows after parturition, is increasingly used as a nutritional supplement to promote gut function and health in other species, including humans. The high levels of whey and casein proteins, immunoglobulins (Igs), and other milk bioactives in BC are adapted to meet the needs of newborn calves. However, BC supplementation may improve health outcomes across other species, especially when immune and gut functions are immature in early life. We provide a review of BC composition and its effects in infants and children in health and selected diseases (diarrhea, infection, growth-failure, preterm birth, necrotizing enterocolitis (NEC), short-bowel syndrome, and mucositis). Human trials and animal studies (mainly in piglets) are reviewed to assess the scientific evidence of whether BC is a safe and effective antimicrobial and immunomodulatory nutritional supplement that reduces clinical complications related to preterm birth, infections, and gut disorders. Studies in infants and animals suggest that BC should be supplemented at an optimal age, time, and level to be both safe and effective. Exclusive BC feeding is not recommended for infants because of nutritional imbalances relative to human milk. On the other hand, adverse effects, including allergies and intolerance, appear unlikely when BC is provided as a supplement within normal nutrition guidelines for infants and children. Larger clinical trials in infant populations are needed to provide more evidence of health benefits when patients are supplemented with BC in addition to human milk or formula. Igs and other bioactive factors in BC may work in synergy, making it critical to preserve bioactivity with gentle processing and pasteurization methods. BC has the potential to become a safe and effective nutritional supplement for several pediatric subpopulations.

## 1. Introduction

Bovine colostrum (BC) is produced by cows in the first days after parturition and provides nutrition and immunological protection of highly sensitive newborn calves [1]. The difference between colostrum and milk results from a partially open blood–milk barrier in the mammary gland around birth [2]. Besides the close link to the mother in which colostrum is produced, many elements of colostrum may have cross-species effects and could be used to support and protect newborns and growing offspring of other species when they are lacking their own mother’s colostrum and/or milk. Here we review the scientific literature related to composition and biological function of BC in pediatric nutrition and gastroenterology, and how this is supported by biomedical animal models of infants and children, most notably the piglet. The topic is timely because there is currently public and scientific debate about the possible risks and benefits of bovine-based milk products for infants and children when human milk is absent or inadequate, particularly for preterm infants and other highly sensitive hospitalized pediatric patients.

As highlighted in the introductory article of this review series [3], the provision of mammary secretions in the form of colostrum and mature milk for newborns is an essential survival function that has evolved over millennia in mammal species. In humans, following the production of colostrum immediately after parturition, the subsequent two weeks postpartum is a time of secretory activation and increased volume secretion of what we eventually consider mature milk [4]. Colostrum serves a vital function for neonates by providing nutrients necessary for energy, growth, and development as they transition from placental supply of elemental nutrients, such as glucose and amino acids, before birth, to oral ingestion of more complex macromolecules, including lactose, proteins, and triglycerides. A second critical function of colostrum and milk after birth is to provide immune protection that supports elements of neonatal innate immunity until maturation of adaptive immunity related to the specific environment [5]. Colostrum functions as a nutritional, immunological, and antimicrobial ‘bridge’ between the mostly sterile fetal life, sustained by the maternal umbilical nutrient supply, and the microbe-rich postnatal environment and enteral breast milk intake. Humans and other mammals often share the same external environment and are exposed to similar microbes, viruses, and fungi along the outer surfaces of the body (skin, lungs, and gut). Consequently, the antimicrobial activity of colostrum from one species should also be (at least partly) effective in another species. The ‘*perinatal colostrum bridge*’ is critical for survival and health for normal newborns in some mammals (e.g., large farm animals with lacking passive immunity transfer before birth), while, in others, the colostrum is important, although not essential for survival (e.g., term human infants).

There are similarities in the composition of colostrum and milk among human and bovine species, but there are also some important differences in both nutrients (carbohydrate, protein, lipid, minerals, and vitamins) and immunological factors, including Igs. Cow’s milk has been used as an important source of supplementary nutrition for infants for centuries in most countries around the world [6]. Beyond the first year of life, other cow’s milk-based foods, such as milk, yogurt, and cheese, are also considered important sources of nutrition in children [7,8]. The potential functional and immunological benefits of dairy products in infants and children, besides nutrition alone, are less clear. In the past decades, the accumulated evidence of clinical benefits of human milk, either mother’s own breast milk or banked donor milk, have made human milk the recommended choice for infant nutrition, especially for preterm infants [9,10,11]. Evidence from preterm infants has raised serious concerns that processed formula products based on cow’s milk lead to more NEC, sepsis (late-onset sepsis, LOS), food intolerance (FI), allergies, and food-protein-induced enterocolitis syndrome (FPIES) in infants fed formula alone, or in combination with human milk [11,12,13,14,15,16]. This has led some clinicians to warn against use of bovine-milk products for all sensitive hospitalized infants [17]. It is unknown if the apparent adverse effects of formula products, relative to mother’s own milk, relate to their bovine origin, the industrial processing steps (i.e., serial heat treatment and filtration steps) and/or addition of vegetable products (i.e., corn-based maltodextrin and vegetable oils), as part of commercial formula production. Whether risk factors for infants differ between term and preterm infants, between bovine products and products from other mammals (donkeys, camels, and goats), and between milk and colostrum is unknown. The possible risks of feeding infants BC are discussed later in this review.

Powdered products containing intact BC, or fractions thereof, have become increasingly available as health foods and their use as nutritional supplements to support gut health for children and adolescents is increasing. Additionally, their utility for newborn infants with limited access to mother’s own milk is currently being investigated. There is considerable speculation about benefits in the marketing of BC products and our goal is to provide a review of the scientific evidence that is used to support these claims. This review highlights the use BC in pediatrics and neonatology, including its use for both as a nutritional and as a preventive or therapeutic supplement for severe pediatric diseases, such as diarrhea, NEC, inflammatory bowel disease (IBD), short-bowel syndrome, and chemotherapy-induced mucositis. Both clinical studies and animal model studies are reviewed and discussed. For detailed insights into BC constituents and applications in adults, readers are referred to other reviews [18,19,20,21] and the companion articles in the present review series in *Nutrients* [3], covering a wide range of possible applications of BC for humans, including gastrointestinal (GI) diseases, immune dysfunctions, and sports medicine.

## 2. Composition and Function of Colostrum in Humans and Animals

### 2.1. Macronutrients

**Figure 1:**
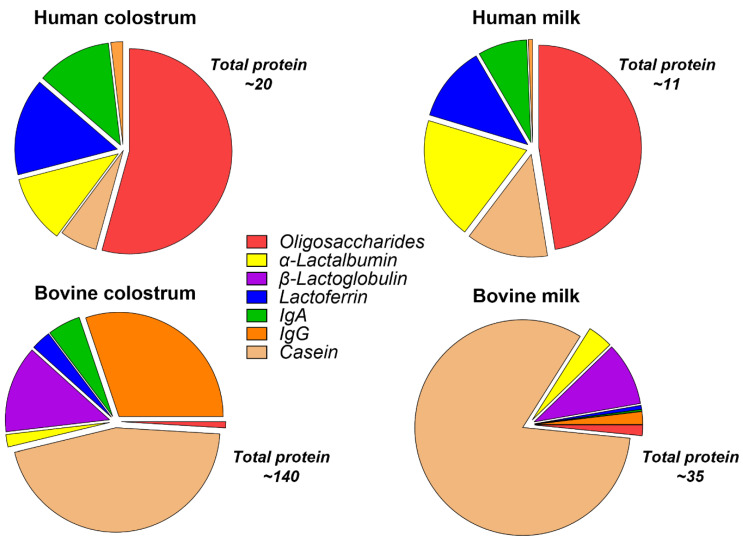
Composition of protein and oligosaccharides in human and bovine colostrum and milk. The figure illustrates the marked differences in total protein content and specific proteins, such as α-lactalbumin, β-lactoglobulin, and casein, between human and bovine colostrum and milk. Human milk and colostrum is rich in α-lactalbumin, LF, and oligosaccharides, relative to bovine milk and colostrum. Conversely, bovine colostrum and milk is rich in casein, β-lactoglobulin, and immunoglobulin G, relative to the human counterparts. All components are expressed as g/L.

Nutritionally, bovine and human colostrum are similar, but they differ mainly in the relative concentration of specific macronutrients. A key feature of BC is its high protein content (~15%) relative to fat (4–6%) and lactose (3 to 4%) [22,23]. The protein and fat contents are higher than lactose in colostrum, but with advancing lactation, the relative content of protein and fat declines and lactose increases. Human and bovine early milk or colostrum contain relatively low amounts of lactose (1.2% and 2–2.9%, respectively), which increase with lactation to 7.0% and 4.8, respectively [24]. The increasing lactose content as milk and the offspring mature suggests an immunological and trophic primary role for colostrum rather than nutritional [4]. The production of lactose increases the movement of water into the secretory vesicles of the mammary epithelium; therefore, low levels of lactose in milk result in increased viscosity. The carbohydrate content of colostrum and milk also comprises oligosaccharides, and these are discussed in more detail in a later section. The protein content of BC and milk is higher than human colostrum and milk (Figure 1), and there are select differences in amino acid content [4,25,26,27,28]. Proteins in human and bovine milk are divided into whey and casein fractions, which comprise differing percentages of the total protein content in mammalian milk across species. In bovine milk, the casein and whey fraction comprise 80% and 20% of the total protein, respectively [29], whereas, in human milk, the ratio is whey predominant (40:60) [30]. However, the proteins within these fractions possess significant homology among species. Among the most widely studied bioactive proteins in colostrum are Igs, lactoferrin (LF), lysozyme, α-lactalbumin, and growth factors. Several casein proteins have also been extensively studied mostly for their role in transporting calcium phosphate and promoting the bioavailability of other milk proteins [31].

The nutritional value of milk proteins in colostrum is a function of the amino acid composition and digestion kinetics through the stomach and upper intestine [32]. Casein proteins form a clot in the stomach that slows release of amino acids for digestion and absorption into the circulation. In contrast, whey proteins are more soluble, in the stomach and empty rapidly into the small intestine for digestion and absorption. These differences between casein and whey protein digestion kinetics have been characterized as the slow vs. fast protein metabolism concept [33] and has relevance for gut motility and metabolism when bovine milk or colostrum products are used for pediatric patients. Susceptibility to gut proteolysis also varies among these proteins, where whey proteins have globular structures that are resistant to proteases, increasing their functional bioactivity throughout the GI tract. The susceptibility of these proteins to GI proteases is also influenced by age; preterm infants have immature gastric acid production and proteolytic digestive capacity. In preterm infants fed breast milk, both of these factors lead to increased survival of intact immunologically important proteins in the intestinal lumen. This latter point suggests that, in term, and especially preterm infants, colostral proteins function not only as a source of amino acids for growth, but also as a source of vital immune protection in the gut. Differences in amino acid availability between human milk and bovine-milk-based infant formulas may induce different plasma amino acid profiles, but also processing effects may affect digestion kinetics, as shown in vitro or in vivo piglet studies [34,35]. 

**Figure 2:**
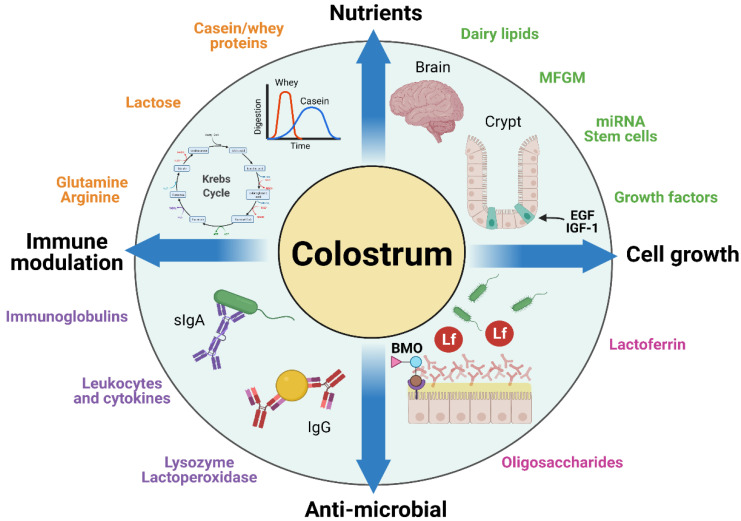
Nutritional and bioactive components present in bovine colostrum. The figure shows some of the key biological functions of bovine colostrum components related to their partially overlapping nutritional, immunomodulatory, antimicrobial, and cell-growth functions. (Created with BioRender.com; accessed on 7 June 2021).

Few studies have tested the possible nutritional benefits of intact BC or fractions thereof in sensitive newborn infants or as prevention or therapy of severe pediatric diseases (see later sections). To understand the potential and limitations of using BC for such conditions beyond the nutritional value of its constituents, it is relevant to briefly review some examples of the numerous components in BC that could play a specific role in pediatric patients. These components can broadly be categorized as having nutritional, growth-stimulating, antimicrobial, and/or antimicrobial effects (Figure 2). A comprehensive review of all the possible bioactive components of BC is presented in the introductory article of this series [3], supplemented with many previous reviews on bioactive constituents in bovine and humane milk with potential effects in infants [36,37,38]. Here, we review five classes of bioactive components relevant to BC in the context of pediatric use: (1) Igs, (2) LF, lysozyme, and α-lactalbumin, (3) milk fat globule membrane proteins, (4) oligosaccharides, and (5) microRNA and stem cells (Figure 2). 

### 2.2. Immunoglobulins

The composition of immune components, such as Igs, LF, and growth factors, is significantly higher in colostrum of most species than in mature milk [4,19,22,39]. Most of these components are well conserved in bovine, porcine, and human milk. Colostral Igs function to provide immune protection within the gut against colonizing microbes and environmental toxins. In many species, they are also the primary transfer of passive immunity. Igs represent a major fraction of colostral protein and the main immune components, including the isotypes IgG, IgA, IgM, and IgD and their subclasses (IgG_1–4_ and IgA_1–2_). There are important differences in the relative composition of these isotypes in human, bovine, and porcine colostrum. Human colostrum is IgA dominant, whereas bovine and porcine colostrum are IgG dominant. IgA occurs as a monomer or dimer, with the latter comprising two IgA molecules joined together by a J-chain and a secretory component. This complex is called secretory IgA (sIgA). In humans, sIgA represents 90% of total immunoglobulin compared with BC, where IgA is only 10% of total Ig. The glycosylation of both the Fc and Fab regions of sIgA play a key role in its protein structure, stability in the gut lumen, and especially its capacity to bind bacterial and host mucosal epitopes [40]. BC and milk contain IgG_1_ and IgG_2_, where IgG_1_ is the main isotype, comprising 70–80% of the immunoglobulin fraction in colostrum. IgG is secreted as a monomer composed of four peptide chains. The concentration of IgG in BC typically ranges from 50 to 100 mg/mL, whereas, in bovine serum, the proportions of IgG_1_ and IgG_2_ are relatively equal, and at lower total concentration (~20 mg/mL). 

**Figure 3:**
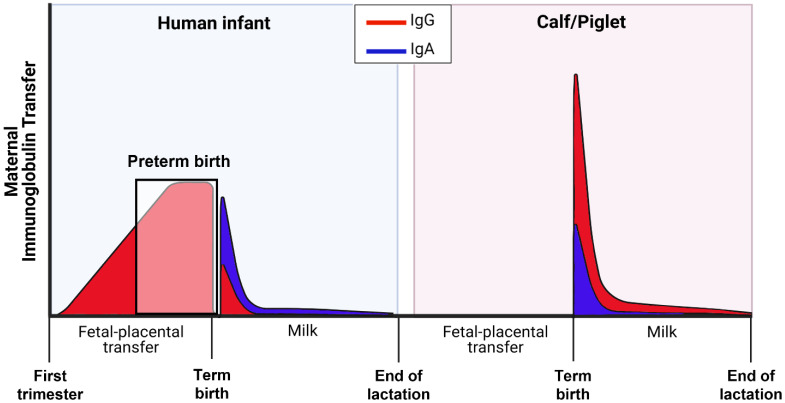
Comparison of maternal immunoglobulin transfer to newborn infants and calves/piglets. The figure illustrates the species differences in the time and mode of maternal immunoglobulin isotype (IgG and IgA) transfer via fetal placental transfer in utero and/or postnatal consumption of colostrum and milk after birth [28,41,42,43,44]. The shaded box shows how premature birth may lead to incomplete maternal fetal–placental transfer of IgG in human infants (created with BioRender.com; accessed on 19 May 2021) [43].

The species-specific necessity for survival and immune protection of newborn mammals depends on whether there is prenatal transfer of maternal Igs (mainly immunoglobulin G, IgG) across the placenta, and/or postnatal transfer across the mucosa of the small intestine (Figure 3). In humans, passive transfer of immunoglobulin occurs largely through prenatal IgG_1_ transfer, mainly in the last trimester of pregnancy 

This may explain why sIgA is the dominant immunoglobulin in colostrum in humans, compared with IgG in domestic animal species. However, in infants born premature, there is incomplete placental IgG transfer and passive immunity that are proportional to the degree of prematurity [45].

Moreover, studies in preterm infants also show increased serum and urinary concentrations of milk proteins, such as sIgA, LF, and α-lactalbumin, compared with term and formula-fed infants [46,47,48]. This suggests that the immature intestine in preterm infants may be more permeable to absorption of macromolecules, such as Igs, in colostrum and mature milk. On the other hand, this increase in intestinal permeability due to immature structural integrity is biologically very different from the highly specialized function of enterocytes in some newborn animals to absorb large molecules by endocytosis with or without involvement of specific (Fc) receptors.

In contrast to infants, large domestic animals (e.g., cattle, pigs, horses, and sheep) lack maternal–placental transfer of Igs and depend almost exclusively on postnatal transfer of maternal IgG via colostrum intake and macromolecule uptake in the newborn gut [49,50,51,52]. Once absorbed into the blood, Igs have relatively slow turnover, and they have a half-life of about 2 weeks [42]. Regardless of species, IgG and sIgA in colostrum serve as a vital first line of defense to neutralize and kill pathogenic microbes in the gut lumen and modulate mucosal immune function to limit inflammation in newborns [19,51].

The capacity for intestinal absorption of colostral immunoglobulin to provide systemic immunity is well established in pigs and calves (see Figure 3). This process occurs via a non-specific endocytotic process, together with active transport via Fc receptors in the mucosal epithelium [53,54]. Importantly, these studies demonstrate the capacity of porcine Fc receptors to transport bovine IgG in cell culture and in vivo. This confirms previous studies showing absorption of bovine IgG in neonatal piglets, albeit at lower rates than porcine IgG [55]. The Fc receptor is expressed in human fetal and neonatal intestine and has been shown to be important for bidirectional transport of IgG across the intestinal epithelium to neighboring dendritic cells for coordination of immune responses to luminal bacteria [56,57,58].

An important consideration regarding the use of BC in humans is whether the IgG present in BC is specific for microbial pathogens present in humans. Considerable evidence shows that bovine IgG can bind to a wide range of pathogenic bacteria, viruses and allergens found in humans [19]. Furthermore, the specificity of bovine and human sIgA isolated from milk is shown to be similar for various pathogenic and commensal bacteria [59]. Thus, the antimicrobial actions of BC products are not restricted to pathogens only present in calves but would likely have effects across a range of microbes in different mammalian species. The situation may be different when it comes to BC interactions with host cells, including the various immune cells and the cells transporting IgG. The fact that BC is able to provide immune protection in piglets, both locally in the gut and systemically via absorbed IgG, suggests that basic functions and transport processes are not species-specific [51,60]. Bovine IgG can also bind to the human Fc receptor with higher affinity than human IgG [61]. The specificity of BC IgG for pathogens mainly present in humans can be optimized by pathogen-specific vaccination of cows prior to collection of milk during lactation to produce ‘hyper-immune colostrum’ [20,62], as discussed further below. The applicability of bovine IgG as a medical supplement has also been developed by using serum-derived bovine immunoglobulin (SBI) that is enriched with >50% IgG. These SBI preparations have been shown to have anti-inflammatory actions on human intestinal epithelial and monocyte cell lines [63]. A few randomized controlled trials in patients with human-immunodeficiency-virus enteropathy and irritable-bowel-syndrome diarrhea, including children, given oral SBI supplements, reported no serious adverse events and modest improvements in GI symptoms [64,65,66,67,68]. In conclusion, the antimicrobial and immunological effects of bovine colostral IgG and IgA are likely to act in both species-specific and species-unspecific ways, depending on the microbe in question and specific host-cell function. 

### 2.3. Lactoferrin, Lysozyme, and α-Lactalbumin

LF is an iron-binding glycoprotein found in many biological secretions but reaches particularly high concentrations in milk. In human colostrum, LF concentration ranges from 5 to 6 mg/mL and decreases to 1 mg/mL in mature milk [69]. In BC, the concentration of LF can range from 1.5 to 5 mg/mL, decreasing to 0.02–0.35 mg/mL in mature cow’s milk [70]. Milk LF has numerous biological functions that range from antioxidant to antitumor and antimicrobial properties. The cellular actions exerted by LF are mediated by the LF receptor (LFR) found in the brush border of the intestinal cell membrane with a sustained abundance in the jejunum throughout the first months of life, as seen in the piglet intestine [71]. After binding to the LFR, LF can be translocated into the cell nucleus and regulate gene transcription, resulting in increased cellular proliferation in the intestine [72]. Beyond its implications in intestinal development, LF’s ability to sequester iron, a necessary source of nutrition for commensal and pathogenic bacteria, contributes to its antimicrobial activity. 

The homology of the human and bovine LF (bLF) amino acid sequences (69% shared amino sequence identity) [73] and the affordability of bLF have made it the most studied LF in both human and animal trials. Several human studies have tested its ability to prevent inflammatory diseases in premature infants, such as NEC and LOS [74]. Randomized controlled trials in preterm infants demonstrated that supplementing infant diet daily with 100 mg/kg/body weight (bw) of bLF (*n* = 472 infants) or 150 mg/kg/bw of recombinant human LF (*n* = 120 infants) protects against NEC and LOS [75]. In contrast, the largest trial (*n* = 2203 infants) of LF in preterm infants (150 mg/kg/day) failed to show any protection against either NEC or LOS [74,76]. These studies formed the basis for recommendations from a Cochrane meta-analysis that included 12 RCTs and 5425 participants given LF supplementation added to enteral feeds in preterm infants. This report found low-certainty evidence that LF supplementation of enteral feeds decreases LOS, but not NEC [77].

The mechanisms through which bLF may protect against LOS and NEC have been investigated in premature piglets. Using a porcine intestinal epithelial cell line, bLF exerted dose-dependent of anti-inflammatory effects on the cells in culture [75]. Additionally, LF’s ability to interact with lipopolysaccharides (LPS) on Gram-negative bacterial cell membrane and compete with LPS for the binding of CD14, a co-receptor for toll-like receptor 4 signaling [78], suggest that it can potentially regulate the host immune response to colonizing microbes. This is particularly interesting given that the pathogenesis of NEC is associated with the premature infant’s response to Gram-negative bacteria through TLR-4 [79]. Studies attempting to confirm the effect of LF on host immune responses to commensal bacteria revealed that splenic and mesenteric lymph-node-derived cells from piglets fed bLF presented an anti-inflammatory cytokine profile before and after ex vivo stimulation with LPS [80]. However, studies in preterm pigs also suggested that the NEC-preventive effects of bLF might be dose-dependent and that high doses of bLF could negatively affect immature epithelial cells via metabolic, apoptotic and inflammatory pathways [81,82]. Collectively, the findings suggest that bLF may function to prevent aberrant inflammatory responses in the intestinal epithelium of newborns. 

Lysozyme, although known to be widely distributed in bodily fluids and present in high concentrations in human breast milk (200–400 µg/mL), is present at a significantly lower concentration in bovine milk (0.05–0.22 µg/mL). Alpha-lactalbumin exists in concentrations ranging from 1.2 to 1.5 mg/mL in bovine milk, has a primary role in the mammary lactose synthesis, and is a source of bioactive peptides and amino acids that support infant growth [83]. Alpha-lactalbumin also is thought to play a role in the development of the infant intestine and brain because of its unique amino acid composition, including tryptophan, lysine, branched-chain amino acids, and sulfur-containing amino acids [84]. Partly replacing the high amounts of β-lactoglobulin in bovine milk with more α-lactalbumin makes the amino acid composition of bovine milk products more similar to that of human milk. However, a recent study in preterm pigs failed to show effects of α-lactalbumin enrichment to a milk diet on growth, gut, immunity, and brain development [85]. Thus, the specific role of BC-derived α-lactalbumin in infant development remains to be elucidated, highlighting the need for further research. Among the rest of the whey fraction components in BC and milk, growth factors are important constituents as possible bioactive components (Figure 2). Many of these growth factors, such as EGF and IGF-1, have been investigated for their isolated roles on gut growth or to prevent disease in infants, children and adults [21,36]. However, these results have been mixed and not been uniformly positive, and only a few of the known bovine milk or colostrum-derived bioactive proteins have been developed for therapeutic use in infants and children. 

### 2.4. Milk Fat Globule Membranes

Milk fat globule (MFG) is a lipid droplet containing triacylglycerols (TAG) that buds from the endoplasmic reticulum into the cytoplasm of mammary gland alveolar epithelial cells [86]. These cytoplasmic lipid droplets are secreted from alveolar epithelial cells by fusing with the plasma membrane, acquiring a peripheral bilayer made of lipids and proteins referred to as the milk fat globule membrane (MFGM). This membrane contains a wide variety of bioactive molecules, such as amphipathic lipids, cerebrosides, gangliosides, mucins, lactadherin, butyrophilin, and glycosylated proteins [87], many of which have been found to have antimicrobial, anti-inflammatory, and anticarcinogenic activities [88]. The contents of MFGM, are highly influenced by environmental factors, as well as lactation and gestation period, maternal genetics, body composition, and diet [86]. Importantly, even though 98% of the milk fat is contained within the MFG, the MFGM constitutes only a minor fraction (1–4%) of the protein content [89] and of total fat (0.2–1%) [88] in human milk (HM) and bovine milk (BM). Bovine and human MFGM have significant structural and functional homology; both are mainly composed of polar lipids that are present in very similar amounts in both species [86,88]. The major groups of lipids building up the MFGM are phospholipids, mainly glycerophospholipids that include phosphatidylcholine (25.2% in HM and 35–36% in BM of total phospholipids), phosphatidylethanolamine (28% in HM and 27–30% in BM), phosphatidylinositol (4.6% in HM and 5–11% in BM), phosphatidylserine (5.9% in HM and 3% in BM), and sphingolipids, which are mainly represented by sphingomyelin (35.7% in HM and 25% in BM). In the same way, the MFGM proteome is highly homologous, and it is composed mainly of adipophilin, butyrophilin, mucin 1, xanthine dehydrogenase/oxidase, mucins, lactadherin, and fatty acid-binding protein, some of which are present in higher amounts in bovine MFGM.

MFGM has been isolated successfully from dairy products, such as BC and cow’s milk, for supplementation in infant formula. The functional role of MFGM has been studied by supplementing milk and formula with MFGM-enriched protein fraction and it is suggested that MFGM protects against infections and modestly impacts the fecal microbiota [90]. Studies performed with premature infants have shown that consumption of sphingomyelin-fortified milk is beneficial for neurobehavioral development [91]. An MFGM-enriched formula diet may improve lipid absorption, availability of essential fatty acids, and therefore neurodevelopment. However, a recent study in preterm pigs failed to show immediate effects of MFGM on the developing brain [92]. Other sphingolipids, such as gangliosides, have also been reported to play critical roles in neurodevelopment and the implications of their dietary consumption have been thoroughly discussed in other reviews [93,94]. Aside from the neurological implications, the digestion of sphingomyelin to sphingosine-1-phosphate (S1P) has been associated with the improvement of intestinal barrier function in vitro by increasing the production and localization of E-cadherin to cell–cell borders [95]. 

The bioactivity of the MFGM proteome ranges from the antimicrobial effects of mucin and xanthine oxidase to the anticancer effects of proteins such as fatty acid binding protein (FABP) [96,97]. Mucins and xanthine oxidases can act as decoy receptors for pathogens in the GI tract, therefore preventing direct bacterial interaction with the epithelium. Additionally, xanthine oxidase’s antimicrobial effects also stem from its ability to generate reactive oxygen and nitrogen species which have bactericidal effects. Other studies have highlighted the benefits of bovine MFGM or MFGM-derived proteins and lipids by demonstrating that MFGM-supplemented formula fed to neonatal rodents improves intestinal growth, Paneth and goblet cell numbers, and tight junction protein patterns to be comparable to those seen in rat pups fed with mother’s milk [98]. In formula-fed infants, bovine MFGM supplementation reduced the levels of *Moraxella catarrhalis*, the leading cause of otitis media in children [99]. Although further studies on the effects of MFGM bioactive components on the health of the human infants are needed, the available literature suggest that BC-derived MFGM may indeed play an important role in infant development.

### 2.5. Oligosaccharides

The major carbohydrates in bovine and human colostrum are lactose and oligosaccharides. Oligosaccharides are composed of more than three monosaccharide [100] units, with a core of lactose or N-acetyl lactosamine, and are classified as neutral or acidic depending on the presence of a sialic acid residue in the molecule [101,102]. Mammalian milks contain a variety of oligosaccharides that consist of different monosaccharides linked to the core lactose of N-acetyl lactosamine [103,104,105,106]. Oligosaccharides are largely undigested in the upper GI tract of the infant and are instead fermented by gut microbes in the distal small intestine and colon [102,107]. Oligosaccharide levels across species are highest in colostrum and decline postpartum [102]. Oligosaccharides are most abundant in humans, ranging from ~7 to 10 g/L, and represent 10% of the total caloric content of mature milk. Human colostrum contains 22–24 g/L oligosaccharides, while BC contains 1 g/L oligosaccharides, and this decreases substantially in the 48 h postpartum [100] (Figure 1). Despite the large differences in oligosaccharide concentrations, bovine and human milk and colostrum oligosaccharides contain similar oligosaccharide structures [108]. It remains unknown if the marked differences in oligosaccharide composition between species (bovine and human) have a specific role or if different species rely on different milk constituents for similar antimicrobial and/or immunomodulatory effects. Regardless, the much higher content of oligosaccharides in human milk versus bovine have led the formula industry to add select oligosaccharides into formula products in attempts to ‘humanize’ infant formula [27].

Milk oligosaccharides have prebiotic activity, anti-adhesion effects, anti-inflammatory properties, and glycome-modifying activity, as well as a role in development of brain and intestinal cells [101]. The supplementation of sialic acid, an oligosaccharide found in both human and bovine colostrum, can improve neural development and memory in piglets [109]. Intact oligosaccharides serve as a prebiotic for bacteria, particularly in the colon [110]. Incubation of HT29 cells with an oligosaccharide found in both human and bovine colostrum resulted in increased adhesion of *Bifidobacterium infantis* [111]. *B. infantis* and other bacteria, growing readily in the presence of human and bovine oligosaccharides, reduce gut pH by producing volatile fatty acids. When epithelial cells were incubated with conditioned media from *Infantis* fermentation, there was a reduction in the release of pro-inflammatory cytokines [112].

Beyond their capacity to drive the colonization of ‘beneficial’ bacterial species in the colon, human and bovine colostrum and milk oligosaccharides may act as competitive inhibitors for pathogenic bacteria binding on the mucosal surface of the small intestine and colon, protecting the neonate from infection [102]. Human milk oligosaccharides (HMOs) have demonstrated anti-infective capabilities against a wide variety of pathogens such as *H. pylori*, *M. meningitides*, and influenza virus in various models [113]. Lane et al. showed that incubation of human epithelial cells with oligosaccharides found in BC reduced the internalization and growth of the devastating diarrheal agent *C. jejuni* [113]. Incubating human colonocytes with the dominant oligosaccharide in BC reduced adhesion of enteropathogenic *E. coli* by 50% [114]. 

Bovine and human milk oligosaccharides (HMO and BMO) have additional immunomodulatory effects on the GI tract itself, beyond their efficacy as prebiotics and pathogen binding agents. When HMO and BMOs are incubated with human epithelial cells, these cells show increased expression of cell surface receptors, chemokines and cytokines, indicating the potential for HMO and BMOs to improve the maturation of the cytokine response in neonates [115]. Ex vivo treatment of cultured peripheral blood mononuclear cells isolated from neonatal piglets with HMOs increased proliferative and anti-inflammatory activity, indicating potential direct immunomodulatory effects of HMO and BMO supplementation on the enteric immune system [116]. Studies in neonatal pigs challenged with rotavirus showed that feeding formula supplemented with HMOs or mixtures of prebiotic oligosaccharides reduced the duration of diarrhea and enhanced T-helper type 1 interferon-gamma and IL-10 cytokines in the ileum [117]. HMO-fed pigs have twice as many natural killer (NK) cells, 36% more mesenteric lymph node effector memory T cells suggesting improved mucosal immune function [118]. Additional work supplementing purified oligosaccharides to neonatal calves receiving heat-treated colostrum demonstrated that oligosaccharides may also increase the absorption of the Igs, potentially improving health and growth outcomes [119]. 

Recent research has investigated whether HMOs contribute to the protective effect of human milk feeding on the incidence of NEC in preterm infants. Rodent models of NEC demonstrated that the protective effects of HMO supplementation are highly HMO structure-specific [120]. These studies identified that disialyllacto-n-tetraose (DSLNT) was the most effective HMO in reducing NEC in rats, whereas a structurally similar and highly abundant HMO, α-1,2-fucosyllactose (2′-FL), has limited effects in both rodents and preterm pigs. The latter studies in preterm pigs also tested whether adding the HMO, 2′-FL, was protective against an enterotoxigenic *Escherichia coli* F18 challenge, because 2′-FL has structural homology to bacterial adhesion sites in the intestine [121,122]. Both of these studies showed that the addition of 2′-FL to formula-fed preterm pigs did not reduce or prevent diarrhea or NEC incidence in the first week of life. A study comparing a mixture of 4 vs. 25 different HMO blends found that these HMOs suppressed intestinal epithelial cell proliferation and had a modest immunomodulatory effect in the intestine in vivo, but did not prevent NEC or diarrhea when added to formula and fed to preterm pigs for 5–11 days after birth. Addition of mixtures of bovine milk oligosaccharides was well tolerated but did not improve any clinical outcomes in preterm pigs [121,122,123,124,125]. In order to move beyond findings in preclinical NEC models, a recent study in matched human mother–infant cohorts correlated HMO composition in infants with healthy and NEC outcomes health [126]. This study showed that that DSLNT concentrations were significantly lower in milk samples from NEC cases compared to controls. Moreover, the lower level of DSLNT was associated with lower relative abundance of *Bifidobacterium longum* and higher relative abundance of *Enterobacter cloacae* in infants with NEC. These studies highlight the specificity of HMOs to modulate NEC and the possible interaction between the immature intestine and developing microbiome community in preterm infants. These findings coupled with the structure and configurational specificity of the beneficial oligosaccharides, indicate that additional work is warranted to explore the implications of milk oligosaccharide supplementation in diets for infants and children, particularly the highly sensitive preterm population. 

### 2.6. Insight into Novel Milk Components

In a continuous effort to explain the benefits of human milk, recent studies have focused on exosomes, their related extracellular vesicles (EV), micro-RNA (miRNA), and stem cells (Figure 2). MicroRNAs are small (18–25 nucleotides) non-coding strands of RNA that exert post-transcriptional regulation on a variety of tissues [127]. They are largely delivered to their target cell or tissue in extracellular vesicles and are found in most secretions from the body, including amniotic fluid, tears, blood, saliva, and milk [128,129]. Moreover, miRNAs in milk and colostrum have two main purposes: first, to maintain the functionality of the mammary gland, and second, to serve as a means of communication between mother and offspring [130]. A majority of miRNAs found in human, bovine, and porcine colostrum are associated with immunological pathways [128,131,132], but other work [130] has demonstrated that miRNAs play an important role in influencing the growth and development of the neonatal GI tract, particularly compared with mature milk.

Extracellular vesicles in human milk and colostrum contain miRNAs associated with immune regulation and metabolism, proteins involved in the signal transduction, and inflammatory response. Milk and colostral extracellular vesicles may represent an anti-inflammatory mechanism underlying the prevention of NEC in preterm infants fed breast milk [133]. Proteomic work has demonstrated that exosomes and extracellular vesicles from BC are enriched with proteins associated with the immune response and growth, indicating their potential role in the regulation of these processes [134]. Co-incubation of bovine colostral vesicles with human macrophage cultures has shown that miRNAs and other proteins packaged in these vesicles have a profound effect on the metabolism, cell migration, and cellular response to LPS challenge [132]. Additionally, Baier et al. [135] showed that in vitro treatment of human cell culture from a variety of tissue origins with miRNAs from BC changes gene expression in human cell culture. Other work has shown that exosomes from bovine milk and BC can both be taken up by human enterocyte culture and maintain the cell cycle in these cells [136].

Although in vitro work has painted a compelling picture of the potential impact of bovine miRNA and exosomes on human tissue, in vivo experiments have been less consistent. The miRNAs packaged in exosomes are largely resistant to degradation in acidic environments, RNAse treatment, desiccation, and freezing [127,132]. Gu et al. [128] demonstrated that miRNAs in extracellular porcine milk and colostrum are generally resistant to harsh conditions and may survive the acidic conditions of the stomach and pass intact into the small intestine. Baier et al. [135] demonstrated that healthy adults absorb miRNAs from mature cow’s milk, and other work has shown that humans may absorb animal- and plant-specific miRNAs. However, it remains unclear if the level of BC-derived absorbed miRNA and the systemic distribution thereof is sufficient to have a meaningful biologic effect, as demonstrated in calves where expression of blood miRNAs correlated poorly with BC consumption [137]. Thus, it is possible that BC-derived miRNAs primarily exert a local effect on the GI tract and not transported to blood in large amounts. 

Stem cells are present in human milk, and they are more abundant in human colostrum than in mature milk [138]. Interestingly, these cells are pluripotent, and work in rodent models has shown that human milk stem cells can be distributed to tissues as varied as the brain, thymus, pancreas, liver, spleen, and kidney. This wide distribution and additional characterization have indicated that there may be a role for these stem cells for regeneration of cells in innate immune system [139,140]. Similar cells have been found in bovine milk and colostrum, but it is unclear if they survive pasteurization and digestion in the human neonate, and how they may be bioactive in the small intestine. Additional research is needed to explore the implications of a pluripotent stem-cell population in BC, particularly if these cells are absorbed and remain pluripotent after absorption. 

Extracellular vesicles, miRNA, and stem cells are all present in measurable amounts in human and bovine colostrum and mature milk. They are potential mechanisms to influence the growth and immune competence of the neonate [137,139,140,141]. However, it remains largely unclear if these effects are species-specific, or if neonatal humans are affected by these components in bovine milk and colostrum. Thus, additional work is required to explore the bioavailability and functions of colostral stem cells, particularly for sensitive neonates. 

## 3. Bovine Colostrum for Growth, Development and Immunity 

### 3.1. Healthy Term Infants and Animal Models 

Limited information is available on the safety and efficacy of providing BC for healthy term infants who have limited or no access to their own mother’s milk immediately after birth. In situations when this happens (e.g., maternal disease, agalactia, or no option to breastfeed), such infants would normally be fed infant formula, or maybe donor human milk if available. For infants beyond 6 months and toddlers beyond the first year of life, commercially available follow-on or growing up formulas have been developed. These formulas were developed to meet the nutritional needs of infants and young toddlers whose complimentary food intake does meet specific nutritional requirements. The American Academy of Pediatrics guidance indicates that follow-up formulas are nutritionally adequate but offer no nutritional advantage over infant formulas [142]. Others have raised questions about the nutritional rationale for follow-on formulas in healthy infants and toddlers [143]. Therefore, in healthy term infants, there would not seem to be a compelling rationale for supplementing BC, especially not in developed countries. 

Over the last decades, many adjustments have been made for conventional formulas to adapt their nutrient contents to age-related needs of infants and children (e.g., less overall protein and casein, more alpha-lactalbumin, and essential oils instead of bovine milk lipids). New ingredients continue to be added to infant formulas in order to make it more similar to that of human milk (‘humanized’ infant formula). More recently, specific components such as LF, osteopontin, lutein, oligosaccharides, MFGMs, and essential fatty acids have been added to specialized formulas [27]. The efforts are based on the assumption that human milk composition is closely adapted to the needs of infants, potentially even matched between individual infants and their own mother. Nevertheless, the biological effects of milk and colostrum may only partly be species-specific and closely matched between mother and offspring in the different species. Thus far, the ability to ‘humanize’ infant formula to accurately reflect nutritive and bioactive constituents of fresh human milk has failed, particularly regarding the highly unstable, heat-sensitive milk bioactive with immunological functions. This lack of progress is especially evident from numerous clinical studies showing the increased risk of diseases (e.g., NEC) in premature infants fed formula compared to human milk. On the other hand, it is possible that healthy term infants are less dependent on optimized levels of nutrients, growth factors, immunomodulatory components, and antimicrobials in human milk and colostrum than preterm, growth-restricted, or diseased infants. The short- and longer-term health benefits of the numerous bioactive factors in fresh human milk or colostrum, and their varying concentrations among individuals remain poorly understood. Regardless, complete substitution of human milk by feeding BC to infants, even for shorter periods, is not recommended, due to the markedly different composition of nutrients and bioactives in BC versus both colostrum and human milk, but more research on dose-response relationships is needed (see later section). 

In human children and adults, some of the effects of bovine IgG are similar to those of IgA in human milk, e.g., binding to human-relevant pathogens, improved phagocytosis mediated through Fc receptors and prevention of infections [19]. In infants, bovine colostral IgG may pass through the gut undigested and have local antimicrobial or immune effects in the gut. Nevertheless, the specificity and effector functions of bovine IgG are not identical to human milk IgA and IgG, and it remains unclear if intact bovine IgG, provided in pure form (isolated from plasma or milk) or as part of BC, can fully or partly restore the lack of mother’s secretory IgA in formula-fed infants. Formula-fed infants receive minimal amounts of immunomodulatory proteins and Igs because processing technologies and heat treatment for microbiological safety normally denature these proteins. The development of more gentle milk-processing technologies in the future may enable inclusion of colostral Igs, or other fractions of BC, into standard infant formula. Longer-term trials are needed to confirm if this reduces GI and respiratory infections, with or without changes to the incidence of allergies and asthma [19]. 

**Figure 4:**
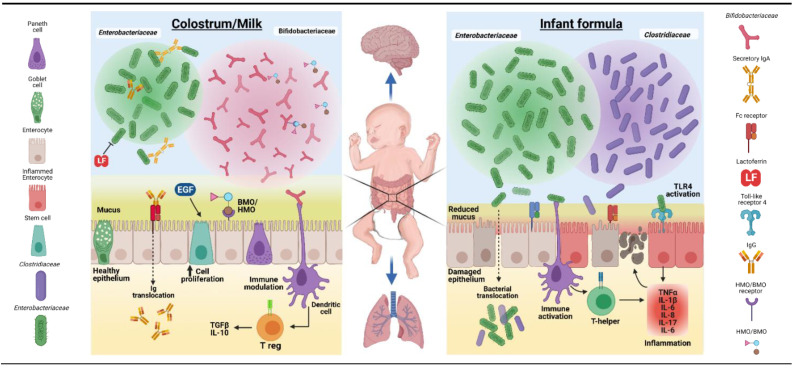
Possible differences between effects of maternal colostrum/milk and formula on the neonatal gut. The figure illustrates some of the proposed functional effects of maternal colostrum/milk bioactive factors on the dominant gut microbiota communities, mucosal epithelium, and immune cells, relative to formula diets. *Enterobacteriaceae*, *Bifidobacteraceae*, and *Clostridiaceae* families represent common bacteria groups found in the developing neonatal gut. The bacterial community composition can be affected by maternal milk factors, such as Igs (IgA and IgG), LF, bovine and human oligosaccharides (BMO/HMO), and a number of growth factors (e.g., EGF). Igs and LF have antimicrobial properties that function to limit epithelial inflammation and apoptosis resulting from activation of Toll-like receptor 4 (TLR4) by *Enterobacteriaceae*. BMO/HMO may serve as a substrate for growth and colonization of *Bifidobacteraceae*, a family of bacteria associated with gut health. The figure also illustrates that key peripheral organs, including the brain and lungs, directly or indirectly may be impacted by colostrum/milk-induced improved gut microbial activity and gut mucosal immune defense. (Created with BioRender.com; accessed on 7 June 2021.)

Bovine colostrum and its components have been extensively investigated for their potential use as a nutritional, growth-stimulating, immunological, and/or antimicrobial supplement for newborns of several animal species when their own maternal milk is deficient or absent. Such animals may even benefit from a short period of exclusive BC feeding, in contrast to infants. Beneficial effects on growth and development have been reported for domestic animals (piglets, foals, and lambs) [42,51,144,145] and some pet-animal species (dogs, cats, and hamsters) [146,147,148,149], either as exclusive diet or supplement. Results from such studies suggest that BC may also be a useful nutritional and bioactive supplement for newborn infants, especially for gut functions, as its biological effects are only partly species-specific. The mechanisms whereby supplementary BC may induce effects in the gut of another species via nutritional, growth-stimulating, immunological, and/or antimicrobial factors (Figure 2) may be similar to possible mechanisms for effects of colostrum/milk versus formula to infants, as illustrated in Figure 4. 

The most widespread use of BC in non-bovine animal species is for piglets. Modern pig breeds often produce more offspring than the number of functional teats on sows, and thus there is a need to provide nutrition and passive immunization of newborn piglets that do not get adequate sow’s colostrum [150]. Supplementing such piglets with intact BC increases their survival, but it remains lower than for species- and herd-specific sow’s colostrum provided via their own mother or foster mothers [144]. As in the human infant, supplementation of BC in piglets has the potential to affect host immunity initially via its interaction with gut pathogens and mucosal epithelial cells. Similarly, supplementation of BC with porcine plasma may further improve GI health and development in newborn pigs, demonstrating that this as a partly species-specific substitution for porcine colostrum, relative to formula [60]. In these studies, the gut trophic and enzyme maturation effects of exclusive BC feeding were similar to, or even exceeded, the effects of porcine colostrum. 

### 3.2. Preterm Infants and Animal Models

In humans, preterm birth (<90% gestation or <37 weeks in humans) occurs in 5–15% of all pregnancies [151]. The most immature, very preterm infants (<32 weeks gestation) suffer most from complications in pregnancy (e.g., inflammation, infection, and placental dysfunction) or immediate postnatal period, resulting in growth failure and various longer-term maladaptation syndromes [152,153]. In the weeks after birth, very preterm infants have high susceptibility to systemic infections (bacteremia and LOS), gut disorders (FI and NEC), lung complications (bronchopulmonary dysplasia, BPD), and brain damage (intraventricular hemorrhage, IVH; cerebral palsy). Not surprisingly, these infants need specialized clinical care, but despite the challenges, survival, health, and growth of preterm infants, even when birth occurs as early as 60–70% gestation (24–28 weeks), have increased dramatically in the past decades. Improved care for these infants has come partly via advances in nutritional care (parenteral nutrition, donor milk, specialized formulas, and better feeding routines) and immunological protection (pro- and antibiotics, and immunomodulatory drugs). Especially in preterm infants, intact BC or fractions thereof may have direct and indirect health effects by modulating the gut immune system, reducing gut inflammation and enhancing mucosal integrity and tissue repair (see Figure 4 for overview of possible mechanisms).

The potential for using BC specifically to support nutrition and immunological protection of this highly sensitive infant population has remained largely unexplored until recently. It is possible that preterm infants, because they have an immature gut, immune system, and metabolism, have some physiological and immunological similarities with newborn farm animals, for whom colostrum intake just after birth is absolutely essential for survival. Recently, stepwise pilot-phase safety trials that involved using a powdered intact BC product during the first 2 weeks of life were completed in Denmark and China [154,155]. 

The studies indicated no adverse clinical effects of BC supplementation, increased enteral protein intake (when feeding with human milk), and/or a shortened time to reach full enteral feeding (when feeding formula). However, elevated plasma tyrosine suggested that excessive protein intake from BC may be a concern, especially in the first week of life (see also later section). These pilot studies suggest that a growth-stimulating effect of adding BC to human milk or formula may relate to indirect effects via the developing gut microbiota, potentially via Igs, which, in turn, affect plasma amino acid levels to a composition more favorable for growth [156]. Studies indicate that intestinal absorption of human colostral proteins can occur [46,47,48,157], yet the capacity of preterm infants to absorb Igs from BC is very limited or absent [154,155]. A larger study is ongoing to confirm these initial observations, feeding a maximum of 50 mL/kg/d BC (ColoDan, Biofiber, Denmark) as a supplement to formula for very preterm infants in the first 2 weeks of life, with time to full enteral feeding as the primary outcome (*n* = 350, ClinicalTrials.gov: NCT03085277). 

The interest to use BC for nutritional and immunological support of preterm infants was sparked by a large series of studies in preterm pigs over 15 years. The neonatal pig has been used as a model for human infant nutrition and gastroenterology for decades based on homologies with regards to physiology, anatomy, and metabolism [158,159,160]. We first began by studying pigs delivered by caesarean section at 90% gestation and observed that feeding porcine colostrum to newborn preterm versus term pigs induced a marked trophic and functional gut response, for some parameters even more than for term animals [161]. Subsequent studies showed that preterm pig intestines were highly sensitive to formula feeding, even more than preterm infants, and spontaneously developed diet- and microbiota-dependent NEC in the first 1 to 2 weeks of life [162,163]. Further studies demonstrated that bovine and porcine colostrum were equally effective in inducing body and gut growth and in protecting against inflammatory conditions in preterm pigs (NEC and LOS; see later disease section [163,164,165,166]). We also showed that BC-fed preterm piglets had a remarkable capacity to rapidly adapt their gut and immune development to that in term pigs (within 1 to 2 weeks) [167,168], while brain and neurodevelopment were slower in reaching normal levels (within 3 to 4 weeks) [169,170,171,172] when fed BC during the first week. These studies clearly indicated the potential to use BC in states of immaturity with a high sensitivity to gut and immune disorders. Importantly, we subsequently showed that human donor milk was also relatively effective in protecting preterm pig against NEC [173]. However, when fed in a minimal enteral feeding protocol, BC was more effective than donor human milk to reduce the density of mucosa-associated bacteria and putative pathogens [174]. These studies in preterm pigs suggest that common component(s) in porcine and bovine colostrum and in human donor milk provide protection against the development of NEC and importantly that these factors are not species-specific. 

The above studies highlight the value of the preterm pig as a preclinical model to evaluate the function and nutritional availability of milk diets, supplements, and novel ingredients in the clinical support of preterm human infants. It is also notable that, besides gut complications (feeding intolerance and NEC), this model incorporates a range of the complications that are commonly known from moderately and very preterm infants, such as respiratory insufficiency; impaired growth; dysmotility; high sepsis sensitivity; metabolic derangements; and kidney, liver, and brain dysfunctions [158,159,175,176]. The integration of all of these complications into the same clinical model is a great advantage over other (rodent) models in neonatology, because any intervention, including the first milk diet, is likely to have multi-organ effects. Due to the similar size (0.6–1 kg) and physiology of 90% gestation preterm pigs and 70% preterm infants, clinical tools and interventions can be made similar (e.g., respiratory care, parenteral/enteral nutrition, use of diagnostic imaging techniques, repeated blood sampling from indwelling catheters, and surgical interventions) [158]. Potentially, preterm pigs may also be used to test the interacting effects of BC for gut and skin healing in preterm newborns and the interacting effects with non-medical clinical routines, such as maternal singing, skin-to-skin contact, and kangaroo care [177]. A recent study demonstrated reduced gut complications in preterm pigs being co-bedded, facilitating sibling skin-to-skin contact [178]. The possibility that the immunological properties of BC may benefit the immature skin of preterm newborns, having altered cell differentiation and perturbed barrier functions [179], remains unexplored. 

### 3.3. Growth-Restricted Infants, Children, or Animal Models

There is a potential to use BC as a supplement in conditions of growth-restriction in developing countries, mainly because the non-nutritive immunoprotective components may alleviate clinical risks of infection and inflammation and thus limit negative impacts on growth at birth or later in infancy and childhood. However, it is not recommended to feed BC as the sole diet to promote growth (see later section). In clinical practice, the specific effects of nutrition-induced growth restriction (e.g., poor placental function before birth and deficient nutrient intake after birth) can be difficult to separate from associated increases in inflammation and infections. A number of studies performed mainly in developed countries indicate that providing hyperimmune supplemental BC reduced severity of diarrhea in children that have evidence of GI infection (see more in later section) [180,181]. Whether colostrum supplementation increases growth and development in growth-restricted infants born at full term, without associated diseases (infectious, inflammatory, or other), is unknown.

For infants, it is well-known that growth restriction at birth is a risk factor for many later diseases, especially if birth also occurs prematurely, making the infant both underweight and immature. A large proportion of preterm infants are born intra-uterine growth restricted (IUGR) and after birth they may continue to experience slow growth related to their increased postnatal complications, resulting in extra-uterine growth restriction (EUGR). Human milk is relatively deficient in some nutrients, especially protein and select minerals (e.g., Ca, P, Fe, and Zn), to support optimal growth of such infants from 1 to 2 weeks of age. Thus, nutrient fortifiers to human milk are needed and a number of products based on typical formula products are available on the market [182]. Due to its high content of both nutrients (protein) and immunomodulatory factors, BC has been speculated to have a potential as a nutrient fortifier to human milk. As a basis for the human trials, we have tested the efficacy of BC as a fortifier to donor human milk in preterm pigs in the first weeks of life. In pigs fed donor human milk, fortification with BC was superior to formula-based fortifiers to support growth, gut function, nutrient absorption, and mucosal defense [183,184]. The studies are significant because they suggest that, even though BC and donor human milk do not contain pig- and species-specific immune components (e.g., Igs and LF), their local gut effects and non-specific systemic immune effects appear sufficient to support health and development in preterm pigs. 

Reduced growth and health challenges of newly weaned piglets can be viewed as an animal model for the nutrition and immune challenges often observed in infants weaned early from their mother and fed alternative milk or vegetable diets, particularly when reared in low-sanitary environments in developing countries. When pigs were weaned later (3 to 4 weeks) on to vegetable-based diets, growth, intestinal function, and immunity were improved (local and systemic IgA, Th1 and Th2 cytokines, and nutrient digestion and absorption) by supplementing small amounts of intact BC or colostrum whey powder (1–10 g/kg body weight per day). Effects occurred mainly during the immediate post-weaning period when digestive complications, microbial perturbations, and adverse immune responses were most pronounced [185,186,187,188,189,190]. A few days of exclusive BC feeding post-weaning (40–45 g/kg/d) reduced diarrhea, *E. coli* (*Enterobacteriaceae*) density in intestinal contents and tissue, and Gram-negative mucosal immune responses (TLR4 and IL-2), but it also increased short chain fatty acid production, in part due to excessive protein supply and fermentation [187,188]. Collectively, the studies in young piglets support that supplemental BC indirectly improves body growth via improved gut functions and immunity, especially in conditions of stress and inflammation, such as the weaning transition, probably reflecting the mechanisms illustrated in Figure 4. As for infants and children, the optimal BC intake to stimulate body growth in various clinical conditions is unclear. 

## 4. Bovine Colostrum to Prevent or Treat Specific Pediatric Diseases

### 4.1. Gut and Lung Infections in Children and Animal Models

One of the most extensively studied applications of BC is treatment of gut infections in children, including rotavirus, enteropathogenic and enterotoxigenic *E. coli, Shigella*, and *Helicobacter pylori* infections (for detailed reviews, see References [19,23,62]). Most of these studies have been conducted by using hyperimmune BC, which is produced by immunizing cows with select pathogens or toxins prior to lactation, with the intention to obtain BC with enriched titers of pathogen- or antigen-specific IgG antibodies. Studies also have used different forms of immunoglobulin-enriched products, including IgG-rich colostrum, IgG-isolates from colostrum or milk, and serum-derived IgG. The products used in the clinical studies were tested for prophylactic or therapeutic effects in field settings, as well as in controlled pathogen challenge models. In two double-blind placebo-controlled studies and two controlled studies designed to treat rotavirus diarrhea, children aged 4–30 months given hyperimmune BC or concentrated antibodies showed significant clinical reductions in duration of diarrhea, stool frequency and duration of virus shedding [191,192,193]. However, a similar study showed only modest benefits [194]. In controlled studies to treat enteropathogenic *E. coli* (EPEC), children with diarrhea were given hyperimmune bovine milk immunoglobulin concentrate for 10 days. Negative EPEC cultures were found in 84% of treated cases, but in only 11% of control children [195]. In another double-blind placebo-controlled study, children with *E. coli*–induced diarrhea were treated with milk concentrate from cows hyperimmunized with enterotoxigenic *E. coli* (ETEC), but no beneficial effects on duration of diarrhea or stool frequency were observed [196]. In other placebo-controlled studies, children infected with *Shighella* were treated with hyperimmune BC with one study showing a reduction in stool frequency [197] and another study no effect [198]. Studies in adults and pathogen challenge studies with *Clostridium difficile*, ETEC, and *Cryptosporidium parvum* showed that hyperimmune BC products can indeed reduce diarrhea and the presence of pathogens in stool [19,62].

Studies designed to prevent gut infections with rotavirus in children have shown a protective effect of feeding hyperimmune BC or immune concentrates [199,200,201]. However, in another large controlled field study, where immune fractions from cows immunized with rotavirus and *E. coli* were added to infant formula, there was no protection against diarrhea [202]. Thus, a summary of the studies in children infected with either rotavirus or *E. coli* suggest that they are not uniformly positive, and are heterogeneous with regard to dose, duration of treatment and form of colostrum product. However, a majority of the studies found a clinical benefit on diarrhea outcomes when treated with hyperimmune BC. Likewise, the studies targeting prevention of rotavirus by feeding hyperimmune BC showed positive effects of reduced diarrhea outcomes. Importantly, hyperimmune BC directed against specific pathogens, in this case rotavirus, has clear therapeutic potentials in children and better effects than against pathogenic bacterial species. A recent meta-analysis including 213 children mostly supplemented with hyperimmune BC indicates reduced severity of diarrhea in children that have evidence of GI infection with *E. coli* and rotavirus [180]. The clinical evidence may suggest that non-immune BC is less effective in controlling gut diseases than hyperimmune colostrum derived from pathogen-specific immunized cows. However, a recent double-blind RCT with 160 children with evidence of GI infection performed in Egypt showed that providing supplemental non-immune BC reduced severity of diarrhea [181]. An important observation from these studies, in children as young as 3 months of age, is the absence of any adverse effects of BC. This point raises the question of whether BC can be safely fed to even younger term infants and those born preterm. Possibly, BC can be particularly effective in preventing bacterial translocation and additional immune protection in immature states of gut microbial colonization (e.g., low species abundance and diversity) and mucosal immunity (e.g., mucous production and immune-cell responses). 

In addition to binding GI tract associated pathogens, IgG from BC can bind to respiratory pathogens, such as human Respiratory Syncytial Virus (RSV), influenza virus and Streptococcus pneumonia [203]. This immunomodulation explains why raw milk consumption (provision of more intact IgG) is associated with fewer upper respiratory tract infections and otitis media [204]. While the studies suggest that BC can prevent upper respiratory tract infections, the many open, non-controlled prospective studies should be interpreted with caution. A role for IgG from BC in preventing or ameliorating viral respiratory tract infections is possible, but whether this protective effect will also affect allergy prevalence remains to be established [19]. The reduced prevalence of allergy in farming families in many countries [205] may be related to intake of unpasteurized milk (containing more intact IgG and immunological factors) by both infants, children as well as their pregnant and lactating mothers.

Again, important lessons are available from studying the effects of intact or fractionated BC in developing pigs with infections. Effects on lung infections are poorly documented, but a number of reports show specific abilities of BC to protect gut epithelial cells against infections, even without prior immunization of pregnant cow’s against specific pig pathogens. Thus, the increased in vitro membrane permeability caused by piglet ETEC bacteria was decreased by three different BC fractions [206]. The fractions contained widely different amounts of Igs and growth factors, suggesting that individual BC factors do not alone explain the protective effects, but antimicrobial compounds such as LF, lysozyme, and lactoperoxidase may all be involved in synergy [206]. In vivo, exclusive feeding with intact BC prevented diarrhea, relative to formula-fed piglets, probably via inducing a higher ratio of lactic acid bacteria to hemolytic *E. Coli*, and lower expression of intestinal Toll-like receptor-4 and IL-2 [187,188]. Similar antibacterial and immune modulating effects in newly weaned piglets were found by other investigators after supplementation with intact BC [185,186,189], but whether such effects extend to organs distant to the gut (e.g., lungs) remains unknown It is noteworthy that small BC supplements to weanling 3-to-4-week-old pigs (0.5–1 g/kg/day) slightly increased systemic IgA levels (likely gut-derived) [185,186], potentially supporting epithelial protection throughout the body, while an exclusive BC diet from day 3 of life (40–45 g/kg/day) had no effects on systemic IgG, IgA, or IgM [188].

### 4.2. Necrotizing Enterocolitis in Preterm Infants and Animal Models

Necrotizing enterocolitis is the leading cause of infant death from GI disease in infants, affecting 3–10% of the hospitalized preterm infants around the world [158,207]. NEC has a mortality as high as 50%, and surgical intervention is necessary in 20–40% of cases, leading to increased morbidity. Three key features necessary for NEC pathogenesis are infant prematurity, the presence of gut microbiota, and enteral feeding, especially infant formula. Importantly, feeding mother’s own breast milk has been shown to effectively reduce the incidence of NEC in preterm infants [9]. The fact that feeding breast milk prevents NEC, especially in preterm infants, suggests that two key features of NEC, namely the microbiota and enteral feeding, may be neutralized by the important immune factors present in human colostrum and milk, but their absence in infant formula. 

A recent meta-analysis summarized results from eight RCT studies (*n* = 394 infants) including mostly preterm infants given human or BC via oropharyngeal route during the first 48 h of life demonstrated no effect on NEC incidence or all-cause mortality, but a trend to reduce culture proven sepsis and reduced time to full feeds [208]. The ongoing large clinical trials on very preterm infants in Denmark and China (<1500 g, total *n* = 700) will soon provide more evidence for safety and efficacy of providing BC to preterm infants to protect against NEC, but also against milder gut immaturity complications, such as constipation, feeding intolerance, and diarrhea [154,155,209]. However, in none of these trials was NEC the primary outcome, because of its low prevalence in the above countries (<5% [207]), requiring a very large sample size to verify NEC effects. Indirect evidence to support BC for protection against NEC comes from studies showing that BC contains IgG and IgA antibodies directed against pathogens that have been associated with NEC, such as *Klebsiella*, *Citrobacter*, *Enterobacter*, and *Serratia* [210]. A pilot clinical study in India (total *n* = 86) showed no benefits of providing a processed BC product to very preterm infants, and there were even indications (although not significant) of increased gut inflammation, indicated by elevated IL-6 in stool samples and radiological features of NEC [211]. Relatively large amounts (5–8 g/kg/d) of product (Pedimmune, Merck, India) were fed for up to 3 weeks with mixed feedings, and its safety related to other constituents than BC alone was questioned (e.g., excessive osmolality). In another recent study on preterm infants (total *n* = 80, <34 weeks gestation), an intact BC product (Immuguard, Dulex-Lab Pharmaceutical, Egypt) increased systemic T-regulatory (Treg) cell number and showed a clear tendency to improved feeding tolerance, growth and resistance to NEC [212]. In this study, maximum 20 mL/kg BC fluid (e.g., <1 g/kg/d) was supplemented to daily formula meals and was compared to formula alone for a maximum of 2 weeks, while gradually transitioning infants to full formula feeding when mother’s own milk was unavailable.

Beyond the few published clinical studies described above, no studies have examined the effects of BC supplementation on NEC. However, there are a number of clinical studies that have tested whether feeding human immunoglobulin preparations can prevent the incidence of NEC [213,214,215,216,217]. Three of the five clinical studies, based on a total of 2095 infants, were reviewed, and it was concluded the current evidence does not support administration of oral immunoglobulin for NEC prevention [218]. The earliest reported studies, showing a protective effect of human Igs against NEC, were small, the outcome assessments not blinded, and infants that received human breast milk were excluded [213,215]. This contrasts with the more recent large randomized placebo-controlled double-blind study where 90% of the infants received breast milk, and with no effect of immunoglobulin supplementation [214]. Here, the immune protection provided by breast milk itself may explain that additional immunoglobulin had no effect. Additionally, Eibl et al. (1988) [213] and Rubaltelli et al. (1991) [215] administered the oral immunoglobulin within the first 24 h following birth, whereas Lawrence et al. (2001) [214] only began oral supplementation after initiation of enteral feeding. The Lawrence et al. (2001) and Rubaltelli et al. (1991) studies used preparations containing predominately IgG, whereas the study by Eibl et al. (1988) used an immunoglobulin mixture containing 73% IgA and 26% IgG. The Lawrence et al. (2001) study also fed a higher dose of immunoglobulin (1200 mg/kg/bw) than the Eibl et al. (1988) and Rubaltelli et al. (1991) studies (600 mg/kg/bw). Thus, from the available studies in preterm infants, is would appear that supplementing BC may be safe, but the benefits on endpoints of growth, morbidity and disease outcomes are lacking given the relatively small number of studies reported. 

The available evidence from pigs suggest that Igs, and a wide range of antibacterial and immunomodulatory factors in BC [3], may work across species, and therefore have protective effects against pathogens and inflammatory reactions in the infant gut. Using preterm pigs as a model of infant NEC [52,158,163,219], BC has been shown to effectively reduce NEC incidence, compared to different feeding regimens of infant formulas, human milk or even amniotic fluid (the natural fetal ‘enteral diet’) [163,164,174,183,184,219,220,221,222,223,224,225,226,227,228,229,230,231,232,233,234,235,236,237,238]. The effects were most pronounced within the first week after preterm birth [174,229,239], with similar efficacy as porcine colostrum [164] and may ameliorate damage induced by earlier or later formula feeding [223,233,239,240], at least when representing a major part of the daily diet [240]. Importantly, BC maintained its in vivo efficacy to protect against NEC in preterm pigs following the spray-drying and heat-pasteurization required for long shelf life, easy handling, and product sterility [232]. Preterm pigs have provided detailed insight into the possible modes of action of BC to prevent NEC development. The effects of BC include stimulation of physical activity [171], feeding-induced mucosal growth [164,223,229], digestive enzyme support [174,184,229,233,240], better nutrient absorption [173,174,224,229,241], improved enteric nervous system development [227,234,242], dampened bacterial overgrowth [163,164,173,174,223,233], reduced intestinal cytokine responses [219,229,230,233], less intestinal permeability [173,174,224,229,233], prevention of pathogen adherence to the gut epithelium [173,184,236], greater production of intestinal mucus [163,243], and reduced bacterial fermentation of nutrients to lactate and short chain fatty acids. It is not clear if BC mediates these effects on NEC by altering gut bacterial colonization. We found no consistent changes in microbiota community profiles associated with BC-induced NEC protection, particularly not during the first week after birth when colonization is chaotic and highly variable [164,173,174,219,223,229,244]. In week two after birth, BC feeding appeared to produce a more robust impact on the gut microbiota, preventing overall bacterial adherence to the mucosa, and proliferation of potentially pathogenic strains of *Campylobacter, Helicobacter*, *Enterococcus*, and some *Clostridia* [174].

### 4.3. Fetal Infection and Neonatal Sepsis in Infants and Animal Models

A large fraction of the infants born prematurely are born in response to maternal infection (chorioamnionitis) with exposure of the developing fetus to gut, lung and systemic inflammation in utero. Such prenatal insults are associated with infections just after birth (early onset sepsis, EOS) and altered immune system development with a range of adverse short- and longer-term outcomes across many organs (e.g., NEC, BPD, and IVH) [245]. The health consequences of fetal inflammation for preterm infants are highly dependent on the type, length and level of fetal infection-induced inflammation, from inducing a precocious (potentially beneficial) immune maturation, to adverse immune defects with subsequent widespread inflammatory insults [246,247]. No studies are available in infants but in theory, BC supplementation may ameliorate the postnatal consequences of fetal inflammation by better control of bacterial colonization after birth and modulation of local and systemic immune responses. In preterm pigs, postnatal BC feeding was clearly more effective than formula feeding to dampen the gut inflammatory effects of experimental intra-uterine infection of the fetus [238,248]. 

Even without fetal inflammatory insults, newborn preterm infants are highly susceptible to LOS) following systemic infections caused by entry of bacteria across their permeable barriers (skin, lung, and gut) or via indwelling catheters for parenteral nutrition and intravenous medication. Between 10 and 40% of hospitalized preterm infants experience one or more periods of systemic infection [183,249,250]. Preterm infants are more predisposed to LOS because of immature function of circulating innate and adaptive immune cells, and an inability to mount an effective immune response to eliminate invading microorganisms. They rely on a disease-tolerant rather than a disease-resistant strategy to combat infections [251]. There is no doubt that mother’s own milk, via IgA and other immunomodulatory components, decreases sepsis sensitivity [252]. 

Whether BC supplementation decreases the risk of sepsis, especially in formula-fed preterm infants, is unclear. A colostrum-like product showed no effect on sepsis in the previously mentioned Indian study [211], while the Egyptian study reported a clear tendency of fewer LOS cases. The latter study showed more systemic Treg cells, inducing a more disease-tolerant state, when formula-fed preterm infants were supplemented with BC from birth [212]. Blood T-regulatory cells (Tregs) are critical factors in regulating the newborn innate and adaptive host response to infection and prevent excessive tissue inflammatory damage whilst allowing development of the immune system components, necessary to combat infections (Th1). The results are promising and may suggest a preventive effect of BC against LOS, but results need to be confirmed in more immature infants receiving variable amounts of human milk and formula. 

In preterm pigs, heat treatment (pasteurization) reduced the capacity of human milk to reduce systemic infection [253], yet the addition of BC to pasteurized donor milk improved its capacity to prevent infection [184]. Direct antimicrobial effects of BC are supported by studies in vitro, both in combination with formula [254] and human milk [250]. Conversely, potential indirect effects, such as improved gut integrity, reduced bacterial translocation, and systemic immunity, in immature newborns are supported by studies in both piglets [229] and rodents [255]. Such luminal effects of BC, facilitating systemic immune protection, may explain why BC feeding markedly reduced adverse clinical symptoms and brain defects in a preterm pig sepsis model [166]. However, reduced systemic neutropenia and bacterial clearance, relative to formula feeding, were observed mainly when BC was fed in the first days after preterm birth, not later [165,239]. Because preterm pigs are able to absorb intact Igs during the first day after birth, some of these effects may be mediated via circulating bovine Igs, and these effects could be pig-specific, and not relevant for human infants. Regardless, enteral feeding with BC may reduce sepsis sensitivity in both infants and pigs, especially just after preterm birth. 

### 4.4. Inflammatory Bowel Disease in Adolescents or Animal Models

Inflammatory bowel disease (IBD) comprises multiple GI disorders, including Crohn’s disease (CD) and ulcerative colitis (UC), and has multifactorial etiology that involves an exaggerated immune response in genetically select groups in response to an environmental factor [256]. The incidence of pediatric IBD is increasing and it develops mainly in adolescence (ages 10–17), but can also occur in younger children less than 5 years of age. Common treatments of pediatric IBD are corticosteroids, amino salicylate, and immunomodulatory agents, such as thiopurines, methotrexate, and anti-TNF antibody drugs. Many of these treatments have important adverse side effects that can reduce body growth and skeletal development during this critical stage of life. Key factors that are commonly altered in cases of IBD and its treatment are the gut microbiota composition and nutrition, since both elements can interact to affect the mucosal immune response [257]. Since nutrition plays a key role in pediatric growth and can also modulate IBD morbidity, dietary approaches have been used in treatment, including use of exclusive and partial enteral nutrition as well as dietary exclusion. The use of exclusive enteral nutrition involves nutrition intervention using a complete liquid formula and is recommended as a first-line therapy for mild-to-moderate IBD by the European Society of Pediatric Gastroenterology, Hepatology and Nutrition (ESPGHAN). 

There are no clinical studies in pediatric patients and only one study that has examined the effect of BC in adult IBD patients with distal colitis [258]. This study involved a small randomized double-blind controlled protocol in middle-aged (45 years) patients with mild to moderately severe distal colitis, given twice daily rectal enemas of either a purified BC product (100 mL of 10% solution) or a control bovine serum albumin solution for four weeks. Several of the patients in both groups were concurrently taking amino salicylate. The BC enemas were well tolerated, and the results showed a significant reduction in clinical symptom score after two weeks in BC-treated patients with no improvement in controls. Further, BC-treated patients’ biopsies showed improvement in histological score. 

The above human study is supported by studies in a mouse DSS colitis model showing that relatively low daily oral gavage of BC (20 mg/kg/bw) improved occult blood, stool consistency, and clinical recovery from colitis, but without preventing the initial weight loss [259]. Conversely, human colostral sIgA (1 to 2 mg/kg/bw) improved recovery of weight loss induced by colitis [259]. Oral gavage with higher daily intakes of BC (100 mg/kg/bw but not 500 mg/kg/bw) in DSS-treated mice also showed protection against tissue injury, and the effects were partially mediated by the IgG present in BC. In a mouse model of TNBS-induced colitis, a relatively high daily oral BC intake (100 mg/mouse) for 21 days reduced colonic tissue injury, proinflammatory cytokine expression, and abundance *E. coli* and *Enterococci* [260]. Taken together, despite only a single human adult IBD study, the results in mouse colitis models suggest that oral BC offers some protection against mucosal inflammation and tissue injury. This experimental evidence encourages further well-designed, clinical trials in pediatric IBD patients to explore the benefits of BC to reduce disease morbidity. 

### 4.5. Short-Bowel Syndrome in Infants or Animal Models

Short bowel syndrome (SBS) is the clinical condition resulting from surgical resection of large portion of intestine due to congenital defects (intestinal atresia and gastroschisis) or disease-associated loss of absorption, leading to an inability to maintain nutrient balance when fed a normal diet. In pediatrics, disease-related SBS is prevalent, resulting from severe NEC lesions, requiring removal the affected (necrotic) parts of the intestine and/or colon. The clinical condition is particularly severe after removal of the distal part of the small intestine, as demonstrated in both patients and animal models, possibly due to the dense localization of immune and enteroendocrine cells in the ileum [261]. 

Preterm infants with SBS after NEC and intestinal resection depend on small volumes of enteral food, in addition to parenteral nutrition, to stimulate gut growth and adaptation, without overloading the immature, remaining parts of the resected gut. Human milk is the recommended diet for all preterm infants, including SBS infants, but when the mother’s own milk or donor human milk is not available, formulas are used. Again, a protein-rich and growth-factor-rich non-human milk diet, such as BC, could be a relevant supplement to this diet. In a pilot study, children (>1 year) with SBS due to a previous episode of NEC and surgery did not improve their intestinal functions (nutrient balance) after inclusion of BC in the enteral diet [262]. Enteral BC supplementation was well tolerated by preterm infants shortly after surgery, with no signs of cow’s milk allergy [263], but more studies are required to substantiate if and how BC should be used for this highly sensitive patient population. A number of both infant- and feeding-related factors (e.g., gestational age, postnatal, resection type, feeding volume, and other diets) may affect responses, making it very difficult to fully document benefits or possible harm in infant studies. 

The clinical complications observed after 50% distal intestine resection in preterm pigs are similar to those in infants subjected to intestinal resection for reasons other than NEC [261]. However, surgical resection in preterm pigs following extensive NEC lesions is difficult due to both practical and ethical limitations in the care of such individuals. Importantly, the postsurgical adaptation responses to enteral formula feeding are reduced in preterm versus term SBS pigs, and the clinical complications, such as hemodynamic instability, hypothermia, intestinal dysmotility, dehydration, respiratory distress, and peritonitis, are more severe [264]. Preterm SBS pigs have a blunted postsurgical increase in intestinal protein synthesis, villus height, crypt depth, and digestive enzyme activities when compared with the corresponding intestinal segment in unresected control pigs. In such piglets, there is limited effect of providing enteral diets, either as infant formula or BC [263], yet the enteroendocrine growth factor glucagon-like peptide 2 (GLP-2) had marked effects immediately after resection [265]. The diminished adaptation in preterm and term newborn pigs contrasts with the structural and functional adaptation of the remnant distal part of the intestine in slightly older (4–8-week-old) suckling pigs that were subjected to resection. In these older pigs, a BC concentrate significantly increased adaptation and circulating levels of insulin-like growth factor 1 (IGF-1) [266,267] and GLP-2 [268]. These findings suggest that the effect of BC in developing SBS individuals is highly dependent on the stage of maturation, as well as many other confounding variables.

### 4.6. Chemotherapy-Induced Mucositis

Childhood leukemia is the most common cancer found in children. Gastrointestinal mucositis is a common adverse advent of cytotoxic anticancer treatment that increase morbidity and mortality. Chemotherapy-induced mucositis (CIM) is an inflammatory process that affects mucosal surfaces and submucosal layers, and is often considered the most serious side effect of cancer treatment. Beyond the problem of mucosal inflammation itself, CIM may adversely affect nutrient and fluid absorption, influence endocrine functions of the intestine, and increase intestinal permeability. CIM also affects innate and adaptive immune functions of the developing intestine, leading to malnutrition, microbial translocation, and systemic inflammatory complications [269]. Considering these complications, coupled with the immune-depressing effects of the cancer itself (especially in leukemia), it is not surprising that BC has been speculated to alleviate the complications of CIM in pediatric patients.

In a recent study on childhood acute lymphoblastic leukemia (ALL), children with newly diagnosed ALL were randomized to receive daily BC or placebo supplements during 4 weeks of chemotherapy induction treatment (total *n* = 62). No differences were found for days with fever (primary outcome), bacteremia, or inflammation, but peak severity of oral mucositis was reduced by BC supplementation, despite that only small amounts of BC (0.5–1 g/kg/d) were ingested by these severely ill pediatric patients [270].

In piglet models of pediatric CIM (without the associated cancer), whole BC had beneficial effects on gut responses after chemotherapy compared with infant formula, but less when compared with intact bovine milk. BC ameliorated the short-term effects of doxorubicin [271,272,273] and also of the more intense myeloablative chemotherapy treatment using busulfan and cyclophosphamide [274,275]. Thus, BC may support the general nutritional status, but it may also promote specific gut mucosal integrity via antimicrobial and endotoxin-neutralizing effects, suppression of gut inflammation, and promotion of mucosal tissue repair. 

## 5. Processing of Bovine Colostrum for Use in Pediatrics 

Infants, especially those born preterm or otherwise immunocompromised, may benefit from the antibacterial, immunomodulatory, and growth-stimulating effects of BC (Figure 2). Therefore, the adverse effects of processing colostrum to its constituents (e.g., bioactives, macronutrients, and micronutrients) are more likely to impact the response of these infants to BC supplementation, relative to more robust term infants and children. Processing procedures differentially reduce the content of many bioactive factors in BC [225,232,254,276], and while this may decrease in vivo bioactivity, it does not eliminate it, partly due to the high amounts present in native BC before processing. Igs and LF are relatively sensitive to heat damage, while other bioactive peptides, such as IGF-1 and TGF-β, are less sensitive [276,277,278]. Consequently, IgG and LF are often used as markers of BC bioactivity. 

There are similar challenges in the preservation of the natural bioactivity of human or bovine milk concentrates. In NEC-sensitive preterm pigs, untreated or gently sterilized (ultraviolet-light lower-temperature sterilization) human donor milk or bovine whey protein concentrates have superior effects to protect against NEC, intestinal inflammation and systemic infections, relative to products subjected to more intense heat [225,253,279,280]. The same may be true for BC. Thus, care should be taken not to eliminate colostral bioactivity during industrial processing, despite the need to provide a microbiologically safe product, for infection-sensitive newborn infants. 

The product- and host-related effects of different processing steps have been extensively studied for dairy products (i.e., freezing, thawing, ultra-filtration, heat- or freeze-drying, food matrix effects, heat pasteurization, ultrahigh heat treatment, irradiation, and storage conditions with regards to temperature, oxygen pressure, and moisture). Much less is known about colostrum, but such processing steps are critical to ensure microbiological safety. The processing effects may differ between mature milk and colostrum due to widely differing composition of both nutrients and heat-sensitive bioactive factors (Figure 3) [3]. Concern for damage to BC by standard heat treatment is not primarily related to macronutrient levels (e.g., protein, lipid, carbohydrate, minerals, and vitamins), but to effects on heat-sensitive proteins that may lose their biological function. Thus, denaturation of colostral proteins by standard heat treatment will not likely reduce amino acid absorption kinetics, which is determined more by protein composition (casein and whey), as shown in piglets [35]. In individuals with immature digestive function (preterm infants), protein digestibility and amino acid absorption may be improved by heat-induced protein denaturation, as shown in infants after ultrahigh temperature (UHT) treatment of formula [281].

Differential clinical responses to BC supplementation of infants and children in the previously reported clinical trials (e.g., gut, immunity, infections, and growth) may arise from processing-related differences in the contents of BC bioactives. Bioactivity of different commercially available BC products varies markedly, as measured in vitro (up to 6-fold) [282]. This inconsistency in bioactivity is a great concern for use of BC as therapy for hospitalized infants and children. The factors in BC that support gut development, immunity and survival in other species are heat-sensitive. When whey protein concentrates are severely heated (80–85 °C, 20–30 min), a reduction in gut-protective effects is observed [279,280]. In contrast, raw, frozen, and mildly heat-treated BC may retain its bioactivity to protect against gut disorders and inflammation, as demonstrated in newborn term pigs [144,224,283,284].

In addition to heat pasteurization, milk and colostrum components are exposed to significant heat during the spray-drying process to obtain a powdered dry product for easy handling and long shelf life. This process, with or without vacuum-concentration, induces relatively minor damage to milk proteins [285], probably due to the large surface area of milk particles during the evaporation and drying process. Both in vitro cell studies and in vivo studies in immature pigs, analyzing fresh and spray-dried BC, support this [224,232,254,276]. Conversely, one or several pasteurization steps, especially using the fast high-temperature method (15 s, 72 °C), relative to the more gentle, slow low-temperature Holder pasteurization method (30 min, 63 °C), induces much greater damage to many colostral proteins [254,276]. In our studies, attempts to secure a near-sterile intact BC product for preterm infants by exposing the product to additional irradiation, induced further protein and amino acid modifications, potentially affecting both nutrient availability and product bioactivity. On the other hand, it can eliminate common low-density pathogens in processed milk powders, such as *Bacillus cereus*, that often escape heat pasteurization steps [254]. While it remains unclear if different processing steps reduce BC bioactivity for infants and children in vivo, it remains critical to balance the need for microbiological safety against processing-related damage to milk proteins. In the ongoing preterm infant studies [154,155,209], we used a low-temperature pasteurized, gamma-irradiated, spray-dried BC product, carefully characterized for its bioactivity in vitro [250,254,276] and in vivo in preterm pigs [232].

The obligation to secure microbiological safety of human donor milk (typically by heat pasteurization) for sensitive preterm newborn infants may decrease its natural protective bioactivity. This may present an option to use BC as a supplement or a fortifier to human donor milk. Pasteurization-induced loss of milk bioactivity will occur for both fluids, but because BC is much richer in many bioactive components, its protective effects may be retained after processing, as shown in preterm pigs studies [232]. Among the available dietary choices for preterm infants, it is also clear that infant formula is inferior in its gut-protective effects to both BC and human donor milk [173]. Alternative methods to secure microbiological safety, such as exposure to ultraviolet light, have been tested, and provide superior quality of human milk for preterm pigs [253], but the general use of this method is still limited. Adverse effects of pasteurization techniques, interacting with long and inappropriate storage conditions (warm, moist, and light), may further accelerate protein modifications that inevitably decrease nutritional value and bioactivity of BC. In recent decades, ultrahigh temperature (UHT)-treated ready-to-feed liquid milk formulas have been widely used for hospitalized infants. Studies indicate that the extra heat-treatment steps for such products, combined with long storage, induce significant damage to milk bioactivity by modifying amino acids and high levels of Maillard reaction to components [286,287]. Ultrahigh-temperature treatment of liquid BC is therefore not a preferred method as part of BC processing for sensitive infants, despite the benefits for easy handling, feeding, and long-term storage.

## 6. Product Consistency and Quality 

Any medical food or nutritional product used in patients, within or outside hospitals, must have a consistent product composition and quality, especially with respect to nutrient and microbiological profiles, but also environmental contaminants. This is a challenge for BC because its composition changes markedly over the first two days of lactation after calving, with rapidly decreasing amounts of many bioactive components [3,277]. Unless the collection time from cows is tightly controlled and restricted, with final products pooled from many farms and cows, this may result in varying BC product compositions, as indicated in a recent survey [282]. In addition, BC composition will vary among different cattle breeds, seasons of the year, cow nutrition, cow parity, and herd health conditions, together affecting product consistency for both nutrients and bioactive constituents. Importantly, it cannot be excluded that cows reared in an environment with pollutants entering the food chain (e.g., heavy metals or persistent organic pollutants, POPs [288]), would also excrete these pollutants in their colostrum. These would likely be excreted in higher amounts in colostrum than in the later mature milk due to the leaky mammary epithelium at parturition [2]. In this perspective, it is critical that colostrum collected from cows treated with antibiotics is tested for antibiotic residues, because any antibiotic residue may be excreted particularly in colostrum. This seems unlikely since most dairies and companies collecting BC for human use need to adhere to strict guidelines for screening antibiotic residues [289]. Thus, bovine colostrum products need to be carefully analyzed to prevent infants and children from exposure to antibiotic residues [290]. Further studies are needed to establish when unwanted components in colostrum, human, or bovine induce health or growth complications short- and longer-term, particularly in sensitive preterm infants [288].

The highly variable production conditions add to the concern for adequate microbiological safety, with possible contamination of the product at farms and/or during processing and packaging (see previous section). Use of BC in pediatrics should normally meet the microbiological safety standards for milk powders. In some countries, microbiological safety standards may be particularly strict for certain microbes when powders are used for sensitive newborn infants (e.g., *E. sakazakii*, *E. coli*, and *Bacillus cereus*). Variable composition, risk of microbiological contaminants, and possible pollutants are factors that require strict quality control with BC products for use in human patients, especially infants and children. In this regard, quality control of BC should be similar to that of infant formula and products derived from human donor milk [291,292,293].

## 7. Possible Health Risks of Using Bovine Colostrum in Pediatrics

Any novel dietary supplement fed to infants and children must be carefully checked for its short- and longer-term safety. Safety concerns regarding BC are particularly relevant for the most vulnerable newborns and children with serious diseases. Some common reservations against use of BC in pediatrics include widely varying product supply, quality, purity and microbiological safety (see previous section), increased child allergy risk, excessive supply of nutrients (protein), nutrient deficiencies (certain amino acids, oligosaccharides, and minerals), excessive supply of growth factors, and inhibition of drug absorption. Most of these concerns are poorly documented in the literature, but it remains important to consider how BC can be used safely, in the optimal formulation and intake levels, at the right time and age, and only in pediatric patients that will in fact benefit from BC supplementation. Several commercial colostrum products are available and were cited in this review and previous reviews [282]. Some products available online are marketed for use in infants and toddlers, but the evidence to support the safety of these products is usually lacking. A commercial bovine colostrum product produced by a US based biotechnology company recently self-affirmed GRAS (Generally Recognized as Safe) after being reviewed by an independent expert panel of scientists. For a substance to be given GRAS determination, scientific data and information about its use and its chemical, toxicological, and microbiological properties must be widely known, and the safety under the conditions of use are approved by consensus among qualified experts.

### 7.1. Cow’s Milk Allergy 

Cow’s milk allergy (CMA) is a very well-studied pediatric condition and affects 2 to 3% of infants in developed countries [14,294] and decreases rapidly after the first years of life. Clinical CMA symptoms are typically seen within few hours of ingesting formula based on cow’s milk and can be IgE or non-IgE mediated. FPIES [16] is part of a larger cluster of non-IgE mediated allergic disease conditions with a prevalence <1%, often related to cow’s milk protein, and affecting the GI tract (e.g., rectal bleeding) [295]. While these allergic conditions in term children may show similarity with NEC in preterm infants, they are likely distinct diseases with limited overlap in predisposing factors and disease mechanisms [15,296]. There is currently no reason to suspect that the risk of developing CMA is greater for BC than for formula or intact cow’s milk. Many bovine milk proteins (>25) can cause allergy and casein subtypes; alpha-lactalbumin and beta-lactoglobulin are potent allergens [297]. These groups are present in similar amounts in BC and milk, while the large amount of non-allergenic immunoglobulin G [298] is present only in BC (Figure 1). 

In our pilot preterm infant studies, bovine immunoglobulin G (bIgG) was not detected in the plasma of preterm infants supplemented with BC [155], and no IgE-mediated allergy was detected for BC-supplemented infants with short-bowel syndrome [263]. Even if bIgG is detected systemically in infants, this would not likely cause allergic reactions. Many human vaccines are developed from Igs raised in other species [42]. In weanling piglets fed BC-rich diets, no anti-bovine immunoglobulin could be detected in plasma although BC modulated the lymphocyte populations locally in the gut [185]. It remains unknown why a commercial IgG-containing BC-like product (6% IgG) supplemented to the diet of preterm infants in India (400 mg IgG/kg/day) was associated with a trend to more gut inflammation [211], but this is not likely to result from bIgG-induced NEC or FPIES symptoms. Preterm infants have an immature immune system that is skewed towards tolerance to foreign proteins, potentially making them more tolerant to introduction of bovine milk, and thereby also colostral proteins from cows, than normal infants. Consequently, the prevalence of CMA in preterm infants tends to be lower, not higher, than for normal infants, indicating that preterm infants are not at increased risk of developing CMA after exposure to BC or milk [299]. Advice against using BC for infants and children, based on CMA concerns, is therefore relevant mainly for individuals already diagnosed with CMA. 

### 7.2. Recommended Safe and Effective Intake Levels

Widely differing intakes of BC have been applied when powdered, whole, or fractionated BC products were supplemented to formula or mother’s own milk for infants and children (0.1–3 g/kg/d, 5–75% of recommended daily protein intake). Typically, the highest intake levels were used in the first week of life for preterm or underweight infants [155,209,211,212], having a deficient supply of own mother’s milk. These infants also have the highest daily protein requirements for metabolism and growth (~4 g/kg/d). Supplementary BC (containing 50–70% protein mainly as IgG) is an attractive way to provide more enteral protein for gut and body growth and metabolism in preterm infants reared on human milk, which is normally considered deficient in protein. However, because the highly enriched IgG may be partly undigested to maintain bioactivity [19], the bioavailability of amino acids in colostrum protein may be less than in highly digestible hydrolyzed protein in infant formulas. This potential issue of amino acid bioavailability of BC proteins highlight a gap in our current knowledge and warrants further investigation, especially in preterm infants with a high protein requirement and an immature digestive capacity.

While a low level of supplementary BC may be nutritionally and immunologically ineffective, excessive BC intake could raise concerns about possible protein toxicity, especially for preterm infants [300]. In such infants, excessive protein may pose a risk for metabolic disturbance, azotemia, and acidosis, in part related to immature metabolic, hepatic, and renal functions. This dysfunction may, in turn, induce brain defects via toxicity of certain amino acids (phenylalanine, glycine, and methionine [301]). In preterm infants, excessive protein supply may first raise plasma levels of an aromatic amino acid, tyrosine. While such feeding-induced ‘hypertyrosinemia’ is not generally considered toxic [302], tyrosine levels may be used as a marker of excessive BC supply to preterm infants during the first weeks after birth [154,155]. Plasma tyrosine levels increase in preterm pigs following BC supplementation during the first week, consistent with the relatively high tyrosine levels in BC [229]. Despite these concerns, amino acid toxicity is a rare condition, even in preterm infants, and normally excess amino acid supply would be effectively excreted as urea [300]. In preterm infants, moderately high blood urea levels (e.g., 5–10 mM) may indicate immature liver and kidney functions and/or excess protein supply, but such urea levels are not toxic, and blood urea level alone is a poor marker of adequate protein intake for growth and protein intake in preterm infants [301]. Collectively, protein toxicity is unlikely to be a problem following BC supplementation to human milk or formula, at least not within the normal upper limits of daily protein intake for infants.

Normal gut motility is diet-dependent and critical to avoid maldigestion, constipation, and diarrhea and GI inflammatory reactions. For sensitive pediatric patients, too much BC-derived casein may adversely affect GI motility and delay gastric emptying due to casein clotting in the stomach. Thus, casein-based pediatric formulas often consist of partly hydrolyzed proteins [303,304] to avoid feeding intolerance, gastric residuals, and constipation, as often observed in preterm infants [305,306,307]. Low-dose BC supplementation within normal protein limits of preterm infants is unlikely to induce casein-related dysmotility and ongoing clinical trials will test this [154,209,224]. In preterm pigs, exclusive BC feeding was associated with accumulation of casein clots, and more gastric mucosal lesions in the first week of life when the gut was still very immature [164,173,174,233]. However, neither casein supplementation to whey-based formula nor rapid advancement of a diet with casein (intact or hydrolyzed) increased the NEC sensitivity in preterm pigs [304,308]. In fact, a moderate BC-induced retention of digesta in the stomach may promote digestion and gut maturation, relative to whey-based formulas, as indicated from food transit studies in preterm pigs [309]. 

The available literature on from infants and children indicate that the optimal time, duration, and intake level of BC supplementation may differ among different clinical conditions, and much research is lacking to define optimal treatments. Pediatric animal models may help to evaluate if excessive or prolonged BC supplementation induces nutritional deficiencies and/or health risks for infants and children. Consistent with its biological role to secure nutrition and immunological protection of newborn calves, there may be limited nutritional benefit of BC when provided in high amounts beyond the first weeks of life although in preterm pigs exclusive BC feeding is effective to promote growth, gut, immunity, and microbiota development beyond 10 days, at least relative to formula [174,239]. In the first week of life, BC supplementation to formula-fed preterm pigs is most NEC-protective when fed in amounts of >50% of the total diet [240], but optimal doses later are less clear, as also shown in chemotherapy-treated term piglets [273]. In a single study, preterm pigs fed exclusive BC in high amounts for seven days showed reduced NEC incidence but abnormal body growth and blood chemistry values (e.g., high blood Na, urea, and lipids) [310]. In all pediatric studies, much less BC was provided than in these animal studies. Relatively high intake levels (8 g/kg/day) of a BC-like highly processed product (6% IgG) for 3 weeks to very preterm infants in India may have led to high osmolality and the reported tendency to adverse NEC effects, but limited product information was provided in the published report [211]. Clearly, more research is required to define when and how much BC, provided in intact or fractionated form and after different processing, should be given to specific pediatric patients to optimize their nutrition, growth, and health outcomes.

## 8. Conclusions

This review provides an extensive overview of the human and animal studies that have examined the potential benefits of feeding BC to infants and children, mainly with regards to its effect in the GI tract. A number of studies in term infants and children have fed BC as a supplement to infant formula or donor human milk with limited adverse effects. More recent studies in preterm infants fed a low supplemental BC intake showed no adverse effects, whereas one study with high intake of a processed BC product, with relatively low IgG levels, produced a tendency to adverse intestinal inflammatory effects. The limited number of studies in infants, and several studies in term and preterm piglets, suggest that feeding exclusive pasteurized BC, or as a supplement to infant formula, protects against several GI diseases, such as rotavirus diarrhea, NEC, sepsis, and CIM mucositis. The evidence suggests benefits and safety especially from hyperimmune BC products directed towards specific pathogens in infants and children. The mechanisms explored to explain the benefits of BC on GI diseases frequently suggest antimicrobial and immune-stimulating functions of Igs, potentially in synergy with other bioactives, such as LF. Nevertheless, it remains unclear how all of these BC bioactive factors act independently or in synergy to promote growth and gut mucosal protection. While safe and effective use of single BC constituents, especially Igs, is reported, the complexity of BC and the variety of clinical conditions provide an argument for using intact BC, rather than fractions or isolated components from BC for infants and children.

The few existing infant studies, together with numerous preclinical animal and cell culture studies, provide a framework to stimulate further investigation of feeding BC in human infants and children. This option has become increasingly possible with the commercial availability of BC preparations to be used as medical foods. This question should also be addressed considering the fact that processed bovine milk products (infant formula) have been fed to infants for >100 years and to very preterm infants for >50 years. Supplementation with BC should only be considered when mother’s own milk or donor human milk are not available or clearly insufficient in nutrients or protective factors. We need safe, effective, and economically viable alternatives to processed infant formulas, which, for preterm infants, is well documented to increase the risk of many diseases, including NEC and sepsis.

It is exceedingly difficult to prove both safety and clear health-promoting effects of new clinical procedures, drugs, or nutritional therapies for hospitalized infants and children. Generally, patient populations are small and the associated complications to test safety and efficacy of new interventions develop with low frequency. Another therapy that has recently been considered for infants and children is probiotics, which only a few decades ago was considered unsafe for immunocompromised preterm infants. Based on the evidence from dozens of randomized controlled trials, many neonatal units are feeding probiotics to hospitalized preterm infants, even though they are not officially approved for such use, in either Europe or United States. The same applies for numerous drugs given to fragile and critically ill infants and children that have not been tested in randomized controlled trials for safety of the intended indication. Given that bovine-milk-based formulas have been given exclusively, or as supplements, to term infants and children for centuries, we suggest that further studies are warranted to test whether pasteurized BC can be a safe and effective nutritional supplement for a number of pediatric patient groups, providing improved immunity and gut health. At the same time, research is needed to better identify the most responsive patient groups and target diseases (e.g., preterm infants with high NEC/sepsis sensitivity, and children with infections and gut complications); the optimal timing, age, and dose of BC; and the optimal BC product for each condition (e.g., optimally processed intact or fractionated BC). 

## References

[1] Hammon H.M., Liermann W., Frieten D., Koch C. (2020). Review: Importance of colostrum supply and milk feeding intensity on gastrointestinal and systemic development in calves. Animal.

[2] Wall S.K., Gross J.J., Kessler E.C., Villez K., Bruckmaier R.M. (2015). Blood-derived proteins in milk at start of lactation: Indicators of active or passive transfer. J. Dairy Sci..

[3] Playford R.J., Weiser M.J. (2021). Bovine Colostrum: Its Constituents and Uses. Nutrients.

[4] Ballard O., Morrow A.L. (2013). Human milk composition: Nutrients and bioactive factors. Pediatr. Clin. North Am..

[5] Goldman A.S. (2002). Evolution of the mammary gland defense system and the ontogeny of the immune system. J. Mammary Gland. Biol. Neoplasia.

[6] Stevens E.E., Patrick T.E., Pickler R. (2009). A history of infant feeding. J. Perinat. Educ..

[7] Riley L.K., Rupert J., Boucher O. (2018). Nutrition in Toddlers. Am. Fam. Physician.

[8] Clark D.C., Cifelli C.J., Pikosky M.A. (2020). Growth and Development of Preschool Children (12–60 Months): A Review of the Effect of Dairy Intake. Nutrients.

[9] Lucas A. (2019). Scientific Evidence for Breastfeeding. Nestle Nutr. Inst. Workshop Ser..

[10] Section on Breastfeeding (2012). Breastfeeding and the use of human milk. Pediatrics.

[11] Maffei D., Schanler R.J. (2017). Human milk is the feeding strategy to prevent necrotizing enterocolitis!. Semin. Perinatol..

[12] Quigley M., Embleton N.D., McGuire W. (2018). Formula versus donor breast milk for feeding preterm or low birth weight infants. Cochrane Database Syst. Rev..

[13] Ananthan A., Balasubramanian H., Rao S., Patole S. (2020). Human Milk-Derived Fortifiers Compared with Bovine Milk-Derived Fortifiers in Preterm Infants: A Systematic Review and Meta-Analysis. Adv. Nutr..

[14] Høst A., Halken S. (2014). Cow’s milk allergy: Where have we come from and where are we going?. Endocr. Metab. Immune Disord. Drug Targets.

[15] Burris A.D., Burris J., Järvinen K.M. (2020). Cow’s Milk Protein Allergy in Term and Preterm Infants: Clinical Manifestations, Immunologic Pathophysiology, and Management Strategies. NeoReviews.

[16] Michelet M., Schluckebier D., Petit L.M., Caubet J.C. (2017). Food protein-induced enterocolitis syndrome—A review of the literature with focus on clinical management. J. Asthma Allergy.

[17] Canvasser J., Hair A.B., Kim J.H., Taylor S.N. (2020). Parent and Provider Perspectives on the Imprecise Label of “Human Milk Fortifier” in the NICU. Nutrients.

[18] Korhonen H., Marnila P., Gill H.S. (2000). Bovine milk antibodies for health. Br. J. Nutr..

[19] Ulfman L.H., Leusen J.H.W., Savelkoul H.F.J., Warner J.O., van Neerven R.J.J. (2018). Effects of Bovine Immunoglobulins on Immune Function, Allergy, and Infection. Front. Nutr..

[20] Rathe M., Müller K., Sangild P.T., Husby S. (2014). Clinical applications of bovine colostrum therapy: A systematic review. Nutr. Rev..

[21] Playford R.J., Macdonald C.E., Johnson W.S. (2000). Colostrum and milk-derived peptide growth factors for the treatment of gastrointestinal disorders. Am. J. Clin. Nutr..

[22] Stelwagen K., Carpenter E., Haigh B., Hodgkinson A., Wheeler T.T. (2009). Immune components of bovine colostrum and milk. J. Anim. Sci..

[23] Hurley W.L., Theil P.K. (2011). Perspectives on immunoglobulins in colostrum and milk. Nutrients.

[24] Puppel K., Golebiewski M., Grodkowski G., Slosarz J., Kunowska-Slosarz M., Solarczyk P., Lukasiewicz M., Balcerak M., Przysucha T. (2019). Composition and Factors Affecting Quality of Bovine Colostrum: A Review. Animals.

[25] Davis T.A., Nguyen H.V., Garcia-Bravo R., Fiorotto M.L., Jackson E.M., Reeds P.J. (1994). Amino acid composition of the milk of some mammalian species changes with stage of lactation. Br. J. Nutr..

[26] Lonnerdal B., Erdmann P., Thakkar S.K., Sauser J., Destaillats F. (2017). Longitudinal evolution of true protein, amino acids and bioactive proteins in breast milk: A developmental perspective. J. Nutr. Biochem..

[27] Ahern G.J., Hennessy A.A., Ryan C.A., Ross R.P., Stanton C. (2019). Advances in Infant Formula Science. Annu. Rev. Food Sci. Technol..

[28] Godden S.M., Lombard J.E., Woolums A.R. (2019). Colostrum Management for Dairy Calves. Vet. Clin. N. Am. Food Anim. Pract..

[29] West D.W.D., Mitchell C.J. (2020). Tracking the Fate of Milk Proteins: Better in Whole or in Part?. J. Nutr..

[30] Martin C.R., Ling P.R., Blackburn G.L. (2016). Review of Infant Feeding: Key Features of Breast Milk and Infant Formula. Nutrients.

[31] Bhat M.Y., Dar T.A., Singh L.R. (2016). Casein Proteins: Structural and Functional Aspects. Milk Proteins—From Structure to Biological Properties and Health Aspects.

[32] Dupont D., Tomé D. (2020). Milk proteins: Digestion and absorption in the gastrointestinal tract. Milk Proteins.

[33] Boirie Y., Dangin M., Gachon P., Vasson M.P., Maubois J.L., Beaufrere B. (1997). Slow and fast dietary proteins differently modulate postprandial protein accretion. Proc. Natl. Acad. Sci. USA.

[34] Bourlieu C., Ménard O., Bouzerzour K., Mandalari G., Macierzanka A., Mackie A.R., Dupont D. (2014). Specificity of Infant Digestive Conditions: Some Clues for Developing Relevant In Vitro Models. Crit. Rev. Food Sci. Nutr..

[35] Welch-Jernigan R.J., Abrahamse E., Stoll B., Smith O., Wierenga P.A., van de Heijning B.J.M., Renes I.B., Burrin D.G. (2019). Postprandial Amino Acid Kinetics of Milk Protein Mixtures are Affected by Composition, But Not Denaturation, in Neonatal Piglets. Curr. Dev. Nutr..

[36] Chatterton D.E., Nguyen D.N., Bering S.B., Sangild P.T. (2013). Anti-inflammatory mechanisms of bioactive milk proteins in the intestine of newborns. Int. J. Biochem. Cell Biol..

[37] Lönnerdal B. (2011). Biological effects of novel bovine milk fractions. Nestle Nutr. Workshop Ser. Paediatr. Program..

[38] Wada Y., Lönnerdal B. (2014). Bioactive peptides derived from human milk proteins—Mechanisms of action. J. Nutr. Biochem..

[39] Korhonen H., Marnila P., Gill H.S. (2000). Milk immunoglobulins and complement factors. Br. J. Nutr..

[40] Cakebread J.A., Humphrey R., Hodgkinson A.J. (2015). Immunoglobulin A in Bovine Milk: A Potential Functional Food?. J. Agric. Food Chem..

[41] Klobasa F., Werhahn E., Butler J.E. (1987). Composition of sow milk during lactation. J. Anim. Sci..

[42] Hedegaard C.J., Heegaard P.M. (2016). Passive immunisation, an old idea revisited: Basic principles and application to modern animal production systems. Vet. Immunol. Immunopathol..

[43] Malek A., Sager R., Kuhn P., Nicolaides K.H., Schneider H. (1996). Evolution of maternofetal transport of immunoglobulins during human pregnancy. Am. J. Reprod. Immunol..

[44] Palmeira P., Quinello C., Silveira-Lessa A.L., Zago C.A., Carneiro-Sampaio M. (2012). IgG placental transfer in healthy and pathological pregnancies. Clin. Dev. Immunol..

[45] Van den Berg J.P., Westerbeek E.A., van der Klis F.R., Berbers G.A., van Elburg R.M. (2011). Transplacental transport of IgG antibodies to preterm infants: A review of the literature. Early Hum. Dev..

[46] Lee J., Kim H.S., Jung Y.H., Choi K.Y., Shin S.H., Kim E.K., Choi J.H. (2015). Oropharyngeal colostrum administration in extremely premature infants: An RCT. Pediatrics.

[47] Goldblum R.M., Schanler R.J., Garza C., Goldman A.S. (1989). Human milk feeding enhances the urinary excretion of immunologic factors in low birth weight infants. Pediatr. Res..

[48] Axelsson I., Jakobsson I., Lindberg T., Polberger S., Benediktsson B., Raiha N. (1989). Macromolecular absorption in preterm and term infants. Acta Paediatr..

[49] Simister N.E. (2003). Placental transport of immunoglobulin G. Vaccine.

[50] Van de Perre P. (2003). Transfer of antibody via mother’s milk. Vaccine.

[51] Sangild P.T. (2003). Uptake of colostral immunoglobulins by the compromised newborn farm animal. Acta Vet. Scandinavica. Suppl..

[52] Sangild P.T. (2006). Gut responses to enteral nutrition in preterm infants and animals. Exp. Biol. Med..

[53] Westrom B., Arevalo Sureda E., Pierzynowska K., Pierzynowski S.G., Perez-Cano F.J. (2020). The Immature Gut Barrier and Its Importance in Establishing Immunity in Newborn Mammals. Front. Immunol..

[54] Stirling C.M., Charleston B., Takamatsu H., Claypool S., Lencer W., Blumberg R.S., Wileman T.E. (2005). Characterization of the porcine neonatal Fc receptor--potential use for trans-epithelial protein delivery. Immunology.

[55] Drew M.D., Owen B.D. (1988). The provision of passive immunity to colostrum-deprived piglets by bovine or porcine serum immunoglobulins. Can. J. Anim Sci..

[56] Pyzik M., Sand K.M.K., Hubbard J.J., Andersen J.T., Sandlie I., Blumberg R.S. (2019). The Neonatal Fc Receptor (FcRn): A Misnomer?. Front. Immunol..

[57] Shah U., Dickinson B.L., Blumberg R.S., Simister N.E., Lencer W.I., Walker W.A. (2003). Distribution of the IgG Fc receptor, FcRn, in the human fetal intestine. Pediatr. Res..

[58] Yoshida M., Kobayashi K., Kuo T.T., Bry L., Glickman J.N., Claypool S.M., Kaser A., Nagaishi T., Higgins D.E., Mizoguchi E. (2006). Neonatal Fc receptor for IgG regulates mucosal immune responses to luminal bacteria. J. Clin. Investig..

[59] Hodgkinson A.J., Cakebread J., Callaghan M., Harris P., Brunt R., Anderson R.C., Armstrong K.M., Haigh B. (2017). Comparative innate immune interactions of human and bovine secretory IgA with pathogenic and non-pathogenic bacteria. Dev. Comp. Immunol..

[60] Jensen A.R., Elnif J., Burrin D.G., Sangild P.T. (2001). Development of intestinal immunoglobulin absorption and enzyme activities in neonatal pigs is diet dependent. J. Nutr..

[61] Nash G.S., MacDermott R.P., Schloemann S., Bertovich M.J., O’Neal J., Porter L., Kulczycki A. (1990). Bovine IgG1, but not IgG2, binds to human B cells and inhibits antibody secretion. Immunology.

[62] Bagwe-Parab S., Yadav P., Kaur G., Tuli H.S., Buttar H.S. (2020). Therapeutic Applications of Human and Bovine Colostrum in the Treatment of Gastrointestinal Diseases and Distinctive Cancer Types: The Current Evidence. Front. Pharmacol..

[63] Detzel C.J., Horgan A., Henderson A.L., Petschow B.W., Warner C.D., Maas K.J., Weaver E.M. (2015). Bovine immunoglobulin/protein isolate binds pro-inflammatory bacterial compounds and prevents immune activation in an intestinal co-culture model. PLoS ONE.

[64] Arrouk R., Herdes R.E., Karpinski A.C., Hyman P.E. (2018). Serum-derived bovine immunoglobulin for children with diarrhea-predominant irritable bowel syndrome. Pediatr. Health Med. Ther..

[65] Asmuth D.M., Hinkle J.E., LaMarca A., Fichtenbaum C.J., Somsouk M., Utay N.S., Shaw A.L., Petschow B.W., Detzel C.J., Weaver E.M. (2017). Evaluation of oral serum-derived bovine immunoglobulins in HIV-infected patients with chronic idiopathic diarrhea. HIV Clin. Trials.

[66] Petschow B.W., Blikslager A.T., Weaver E.M., Campbell J.M., Polo J., Shaw A.L., Burnett B.P., Klein G.L., Rhoads J.M. (2014). Bovine immunoglobulin protein isolates for the nutritional management of enteropathy. World J. Gastroenterol..

[67] Petschow B.W., Burnett B., Shaw A.L., Weaver E.M., Klein G.L. (2014). Serum-derived bovine immunoglobulin/protein isolate: Postulated mechanism of action for management of enteropathy. Clin. Exp. Gastroenterol..

[68] Van Arsdall M., Haque I., Liu Y., Rhoads J.M. (2016). Is There a Role for the Enteral Administration of Serum-Derived Immunoglobulins in Human Gastrointestinal Disease and Pediatric Critical Care Nutrition?. Adv. Nutr..

[69] Montagne P., Culliere M.L., Bene M.C., Faure G. (2001). Changes in lactoferrin and lysozyme levels in human milk during the first twelve weeks of lactation. Bioactive Components of Human Milk.

[70] Donovan S.M., Odle J. (1994). Growth factors in milk as mediators of infant development. Annu. Rev. Nutr..

[71] Gislason J., Iyer S., Hutchens T.W., Lonnerdal B. (1993). Lactoferrin receptors in piglet small intestine: Lactoferrin binding properties, ontogeny, and regional distribution in the gastrointestinal tract. J. Nutr. Biochem..

[72] Donovan S.M. (2016). The Role of Lactoferrin in Gastrointestinal and Immune Development and Function: A Preclinical Perspective. J. Pediatr..

[73] Zhang J.L., Han X., Shan Y.J., Zhang L.W., Du M., Liu M., Yi H.X., Ma Y. (2018). Effect of bovine lactoferrin and human lactoferrin on the proliferative activity of the osteoblast cell line MC3T3-E1 in vitro. J. Dairy Sci..

[74] Embleton N.D., Berrington J.E. (2020). Clinical Trials of Lactoferrin in the Newborn: Effects on Infection and the Gut Microbiome. Nestle Nutr. Inst. Workshop Ser..

[75] Manzoni P., Meyer M., Stolfi I., Rinaldi M., Cattani S., Pugni L., Romeo M.G., Messner H., Decembrino L., Laforgia N. (2014). Bovine lactoferrin supplementation for prevention of necrotizing enterocolitis in very-low-birth-weight neonates: A randomized clinical trial. Early Hum. Dev..

[76] Manzoni P., Dall’Agnola A., Tomé D., Kaufman D.A., Tavella E., Pieretto M., Messina A., De Luca D., Bellaiche M., Mosca A. (2018). Role of Lactoferrin in Neonates and Infants: An Update. Am. J. Perinatol..

[77] Pammi M., Suresh G. (2020). Enteral lactoferrin supplementation for prevention of sepsis and necrotizing enterocolitis in preterm infants. Cochrane Database Syst. Rev..

[78] Actor J.K., Hwang S., Kruzel M.L. (2009). Lactoferrin as a natural immune modulator. Curr. Pharm. Des..

[79] Afrazi A., Sodhi C.P., Richardson W., Neal M., Misty G., Siggers R., Hackman D.J. (2011). New insights into pathogenesis and treatment of necrotizing enterocolitis: Toll-like receptors and beyond. Pediatr. Res..

[80] Comstock S.S., Reznikov E.A., Contractor N., Donovan S.M. (2014). Dietary bovine lactoferrin alters mucosal and systemic immune cell responses in neonatal piglets. J. Nutr..

[81] Nguyen D.N., Jiang P., Stensballe A., Bendixen E., Sangild P.T., Chatterton D.E. (2016). Bovine lactoferrin regulates cell survival, apoptosis and inflammation in intestinal epithelial cells and preterm pig intestine. J. Proteom..

[82] Nguyen D.N., Li Y., Sangild P.T., Bering S.B., Chatterton D.E. (2014). Effects of bovine lactoferrin on the immature porcine intestine. Br. J. Nutr..

[83] Layman D.K., Lonnerdal B., Fernstrom J.D. (2018). Applications for alpha-lactalbumin in human nutrition. Nutr. Rev..

[84] Krissansen G.W. (2007). Emerging health properties of whey proteins and their clinical implications. J. Am. Coll. Nutr..

[85] Nielsen C.H., Hui Y., Nguyen D.N., Ahnfeldt A.M., Burrin D.G., Hartmann B., Heckmann A.B., Sangild P.T., Thymann T., Bering S.B. (2020). Alpha-Lactalbumin Enriched Whey Protein Concentrate to Improve Gut, Immunity and Brain Development in Preterm Pigs. Nutrients.

[86] Lee H., Padhi E., Hasegawa Y., Larke J., Parenti M., Wang A., Hernell O., Lonnerdal B., Slupsky C. (2018). Compositional Dynamics of the Milk Fat Globule and Its Role in Infant Development. Front. Pediatr..

[87] Koletzko B. (2016). Human Milk Lipids. Ann. Nutr. Metab..

[88] Manoni M., Di Lorenzo C., Ottoboni M., Tretola M., Pinotti L. (2020). Comparative Proteomics of Milk Fat Globule Membrane (MFGM) Proteome across Species and Lactation Stages and the Potentials of MFGM Fractions in Infant Formula Preparation. Foods.

[89] Brink L.R., Lonnerdal B. (2020). Milk fat globule membrane: The role of its various components in infant health and development. J. Nutr. Biochem..

[90] Hernell O., Lonnerdal B., Timby N. (2020). Milk Fat Globule Membranes: Effects on Microbiome, Metabolome, and Infections in Infants and Children. Nestle Nutr. Inst. Workshop Ser..

[91] Tanaka K., Hosozawa M., Kudo N., Yoshikawa N., Hisata K., Shoji H., Shinohara K., Shimizu T. (2013). The pilot study: Sphingomyelin-fortified milk has a positive association with the neurobehavioural development of very low birth weight infants during infancy, randomized control trial. Brain Dev..

[92] Henriksen N.L., Aasmul-Olsen K., Venkatasubramanian R., Nygaard M.K.E., Sprenger R.R., Heckmann A.B., Ostenfeld M.S., Ejsing C.S., Eskildsen S.F., Müllertz A. (2021). Dairy-Derived Emulsifiers in Infant Formula Show Marginal Effects on the Plasma Lipid Profile and Brain Structure in Preterm Piglets Relative to Soy Lecithin. Nutrients.

[93] Palmano K., Rowan A., Guillermo R., Guan J., McJarrow P. (2015). The role of gangliosides in neurodevelopment. Nutrients.

[94] Rueda R., Maldonado J., Narbona E., Gil A. (1998). Neonatal dietary gangliosides. Early Hum. Dev..

[95] Greenspon J., Li R., Xiao L., Rao J.N., Sun R., Strauch E.D., Shea-Donohue T., Wang J.Y., Turner D.J. (2011). Sphingosine-1-phosphate regulates the expression of adherens junction protein E-cadherin and enhances intestinal epithelial cell barrier function. Dig. Dis. Sci..

[96] Hernell O., Timby N., Domellof M., Lonnerdal B. (2016). Clinical Benefits of Milk Fat Globule Membranes for Infants and Children. J. Pediatr..

[97] Spitsberg V.L. (2005). *Invited Review*: Bovine Milk Fat Globule Membrane as a Potential Nutraceutical. J. Dairy Sci..

[98] Bhinder G., Allaire J.M., Garcia C., Lau J.T., Chan J.M., Ryz N.R., Bosman E.S., Graef F.A., Crowley S.M., Celiberto L.S. (2017). Milk Fat Globule Membrane Supplementation in Formula Modulates the Neonatal Gut Microbiome and Normalizes Intestinal Development. Sci. Rep..

[99] Timby N., Domellof M., Holgerson P.L., West C.E., Lonnerdal B., Hernell O., Johansson I. (2017). Oral Microbiota in Infants Fed a Formula Supplemented with Bovine Milk Fat Globule Membranes—A Randomized Controlled Trial. PLoS ONE.

[100] Urashima T., Taufik E., Fukuda K., Asakuma S. (2013). Recent advances in studies on milk oligosaccharides of cows and other domestic farm animals. Biosci. Biotechnol. Biochem..

[101] Albrecht S., Lane J.A., Mariño K., Al Busadah K.A., Carrington S.D., Hickey R.M., Rudd P.M. (2014). A comparative study of free oligosaccharides in the milk of domestic animals. Br. J. Nutr..

[102] Gopal P.K., Gill H.S. (2000). Oligosaccharides and glycoconjugates in bovine milk and colostrum. Br. J. Nutr..

[103] Donovan S.M., Comstock S.S. (2016). Human Milk Oligosaccharides Influence Neonatal Mucosal and Systemic Immunity. Ann. Nutr. Metab..

[104] Pacheco A.R., Barile D., Underwood M.A., Mills D.A. (2015). The impact of the milk glycobiome on the neonate gut microbiota. Annu. Rev. Anim. Biosci..

[105] Smilowitz J.T., Lebrilla C.B., Mills D.A., German J.B., Freeman S.L. (2014). Breast milk oligosaccharides: Structure-function relationships in the neonate. Annu. Rev. Nutr..

[106] Bode L., Jantscher-Krenn E. (2012). Structure-function relationships of human milk oligosaccharides. Adv. Nutr. (Bethesda Md.).

[107] Newburg D.S. (2009). Neonatal protection by an innate immune system of human milk consisting of oligosaccharides and glycans. J. Anim. Sci..

[108] Aldredge D.L., Geronimo M.R., Hua S., Nwosu C.C., Lebrilla C.B., Barile D. (2013). Annotation and structural elucidation of bovine milk oligosaccharides and determination of novel fucosylated structures. Glycobiology.

[109] Wang B., Yu B., Karim M., Hu H., Sun Y., McGreevy P., Petocz P., Held S., Brand-Miller J. (2007). Dietary sialic acid supplementation improves learning and memory in piglets. Am. J. Clin. Nutr..

[110] Harmsen H.J., Wildeboer-Veloo A.C., Raangs G.C., Wagendorp A.A., Klijn N., Bindels J.G., Welling G.W. (2000). Analysis of intestinal flora development in breast-fed and formula-fed infants by using molecular identification and detection methods. J. Pediatr. Gastroenterol. Nutr..

[111] Morrin S.T., Lane J.A., Marotta M., Bode L., Carrington S.D., Irwin J.A., Hickey R.M. (2019). Bovine colostrum-driven modulation of intestinal epithelial cells for increased commensal colonisation. Appl. Microbiol. Biotechnol..

[112] Davis E.C., Wang M., Donovan S.M. (2017). The role of early life nutrition in the establishment of gastrointestinal microbial composition and function. Gut Microbes.

[113] Lane J.A., Mariño K., Naughton J., Kavanaugh D., Clyne M., Carrington S.D., Hickey R.M. (2012). Anti-infective bovine colostrum oligosaccharides: Campylobacter jejuni as a case study. Int. J. Food Microbiol..

[114] Angeloni S., Ridet J.L., Kusy N., Gao H., Crevoisier F., Guinchard S., Kochhar S., Sigrist H., Sprenger N. (2005). Glycoprofiling with micro-arrays of glycoconjugates and lectins. Glycobiology.

[115] Lane J.A., O’Callaghan J., Carrington S.D., Hickey R.M. (2013). Transcriptional response of HT-29 intestinal epithelial cells to human and bovine milk oligosaccharides. Br. J. Nutr..

[116] Comstock S.S., Wang M., Hester S.N., Li M., Donovan S.M. (2014). Select human milk oligosaccharides directly modulate peripheral blood mononuclear cells isolated from 10-d-old pigs. Br. J. Nutr..

[117] Li M., Monaco M.H., Wang M., Comstock S.S., Kuhlenschmidt T.B., Fahey G.C., Miller M.J., Kuhlenschmidt M.S., Donovan S.M. (2014). Human milk oligosaccharides shorten rotavirus-induced diarrhea and modulate piglet mucosal immunity and colonic microbiota. ISME J..

[118] Comstock S.S., Li M., Wang M., Monaco M.H., Kuhlenschmidt T.B., Kuhlenschmidt M.S., Donovan S.M. (2017). Dietary Human Milk Oligosaccharides but Not Prebiotic Oligosaccharides Increase Circulating Natural Killer Cell and Mesenteric Lymph Node Memory T Cell Populations in Noninfected and Rotavirus-Infected Neonatal Piglets. J. Nutr..

[119] Short D.M., Moore D.A., Sischo W.M. (2016). A Randomized Clinical Trial Evaluating the Effects of Oligosaccharides on Transfer of Passive Immunity in Neonatal Dairy Calves. J. Vet. Intern. Med..

[120] Bode L. (2018). Human Milk Oligosaccharides in the Prevention of Necrotizing Enterocolitis: A Journey from in vitro and in vivo Models to Mother-Infant Cohort Studies. Front. Pediatr..

[121] Cilieborg M.S., Sangild P.T., Jensen M.L., Østergaard M.V., Christensen L., Rasmussen S.O., Mørbak A.L., Jørgensen C.B., Bering S.B. (2017). α1,2-Fucosyllactose Does Not Improve Intestinal Function or Prevent Escherichia coli F18 Diarrhea in Newborn Pigs. J. Pediatr. Gastroenterol. Nutr..

[122] Cilieborg M.S., Bering S.B., Østergaard M.V., Jensen M.L., Krych Ł., Newburg D.S., Sangild P.T. (2016). Minimal short-term effect of dietary 2′-fucosyllactose on bacterial colonisation, intestinal function and necrotising enterocolitis in preterm pigs. Br. J. Nutr..

[123] Obelitz-Ryom K., Rendboe A.K., Nguyen D.N., Rudloff S., Brandt A.B., Nielsen D.S., Heckmann A.B., Chichlowski M., Sangild P.T., Thymann T. (2018). Bovine Milk Oligosaccharides with Sialyllactose for Preterm Piglets. Nutrients.

[124] Rasmussen S.O., Martin L., Østergaard M.V., Rudloff S., Roggenbuck M., Nguyen D.N., Sangild P.T., Bering S.B. (2017). Human milk oligosaccharide effects on intestinal function and inflammation after preterm birth in pigs. J. Nutr. Biochem..

[125] Bering S.B. (2018). Human Milk Oligosaccharides to Prevent Gut Dysfunction and Necrotizing Enterocolitis in Preterm Neonates. Nutrients.

[126] Masi A.C., Embleton N.D., Lamb C.A., Young G., Granger C.L., Najera J., Smith D.P., Hoffman K.L., Petrosino J.F., Bode L. (2020). Human milk oligosaccharide DSLNT and gut microbiome in preterm infants predicts necrotising enterocolitis. Gut.

[127] Kim S.Y., Yi D.Y. (2020). Components of human breast milk: From macronutrient to microbiome and microRNA. Clin. Exp. Pediatr..

[128] Gu Y., Li M., Wang T., Liang Y., Zhong Z., Wang X., Zhou Q., Chen L., Lang Q., He Z. (2012). Lactation-related microRNA expression profiles of porcine breast milk exosomes. PLoS ONE.

[129] Zempleni J., Sukreet S., Zhou F., Wu D., Mutai E. (2019). Milk-Derived Exosomes and Metabolic Regulation. Annu. Rev. Anim. Biosci..

[130] Van Hese I., Goossens K., Vandaele L., Opsomer G. (2020). Invited review: MicroRNAs in bovine colostrum—Focus on their origin and potential health benefits for the calf. J. Dairy Sci..

[131] Özdemir S. (2020). Identification and comparison of exosomal microRNAs in the milk and colostrum of two different cow breeds. Gene.

[132] Sun Q., Chen X., Yu J., Zen K., Zhang C.Y., Li L. (2013). Immune modulatory function of abundant immune-related microRNAs in microvesicles from bovine colostrum. Protein Cell.

[133] O’Reilly D., Dorodnykh D., Avdeenko N.V., Nekliudov N.A., Garssen J., Elolimy A.A., Petrou L., Simpson M.R., Yeruva L., Munblit D. (2021). Perspective: The Role of Human Breast-Milk Extracellular Vesicles in Child Health and Disease. Adv. Nutr..

[134] Samuel M., Chisanga D., Liem M., Keerthikumar S., Anand S., Ang C.S., Adda C.G., Versteegen E., Jois M., Mathivanan S. (2017). Bovine milk-derived exosomes from colostrum are enriched with proteins implicated in immune response and growth. Sci. Rep..

[135] Baier S.R., Nguyen C., Xie F., Wood J.R., Zempleni J. (2014). MicroRNAs are absorbed in biologically meaningful amounts from nutritionally relevant doses of cow milk and affect gene expression in peripheral blood mononuclear cells, HEK-293 kidney cell cultures, and mouse livers. J. Nutr..

[136] Ross M., Atalla H., Karrow N., Mallard B.A. (2020). The bioactivity of colostrum and milk exosomes of high, average, and low immune responder cows on human intestinal epithelial cells. J. Dairy Sci..

[137] Kirchner B., Buschmann D., Paul V., Pfaffl M.W. (2020). Postprandial transfer of colostral extracellular vesicles and their protein and miRNA cargo in neonatal calves. PLoS ONE.

[138] Goudarzi N., Shabani R., Ebrahimi M., Baghestani A., Dehdashtian E., Vahabzadeh G., Soleimani M., Moradi F., Katebi M. (2020). Comparative phenotypic characterization of human colostrum and breast milk-derived stem cells. Hum. Cell.

[139] Ghosh A. (2020). Breast Milk Stem Cell Survival in Neonate’s Gut, Entery into Neonate Circulation and Adaption by the Body. Curr. Stem Cell Res. Ther..

[140] Cacho N.T., Lawrence R.M. (2017). Innate Immunity and Breast Milk. Front. Immunol..

[141] Admyre C., Johansson S.M., Qazi K.R., Filén J.J., Lahesmaa R., Norman M., Neve E.P., Scheynius A., Gabrielsson S. (2007). Exosomes with immune modulatory features are present in human breast milk. J. Immunol..

[142] Martinez J.A., Ballew M.P. (2011). Infant formulas. Pediatr. Rev..

[143] Przyrembel H., Agostoni C. (2013). Growing-up milk: A necessity or marketing?. World Rev. Nutr. Diet..

[144] Gomez G.G., Phillips O., Goforth R.A. (1998). Effect of immunoglobulin source on survival, growth, and hematological and immunological variables in pigs. J. Anim. Sci..

[145] Staak C. (1992). Bovine colostrum and protection of young animals. Berl. Munch. Tierarztl. Wochenschr..

[146] Heidebrecht H.J., Weiss W.J., Pulse M., Lange A., Gisch K., Kliem H., Mann S., Pfaffl M.W., Kulozik U., von Eichel-Streiber C. (2019). Treatment and Prevention of Recurrent Clostridium difficile Infection with Functionalized Bovine Antibody-Enriched Whey in a Hamster Primary Infection Model. Toxins.

[147] Lyerly D.M., Bostwick E.F., Binion S.B., Wilkins T.D. (1991). Passive immunization of hamsters against disease caused by Clostridium difficile by use of bovine immunoglobulin G concentrate. Infect. Immun..

[148] Satyaraj E., Reynolds A., Pelker R., Labuda J., Zhang P., Sun P. (2013). Supplementation of diets with bovine colostrum influences immune function in dogs. Br. J. Nutr..

[149] Casal M.L., Jezyk P.F., Giger U. (1996). Transfer of colostral antibodies from queens to their kittens. Am. J. Vet. Res..

[150] Kirkden R.D., Broom D.M., Andersen I.L. (2013). Invited review: Piglet mortality: Management solutions. J. Anim. Sci..

[151] Blencowe H., Cousens S., Chou D., Oestergaard M., Say L., Moller A.B., Kinney M., Lawn J. (2013). Born too soon: The global epidemiology of 15 million preterm births. Reprod. Health.

[152] Lawn J.E., Blencowe H., Waiswa P., Amouzou A., Mathers C., Hogan D., Flenady V., Frøen J.F., Qureshi Z.U., Calderwood C. (2016). Stillbirths: Rates, risk factors, and acceleration towards 2030. Lancet.

[153] Lee A.C., Katz J., Blencowe H., Cousens S., Kozuki N., Vogel J.P., Adair L., Baqui A.H., Bhutta Z.A., Caulfield L.E. (2013). National and regional estimates of term and preterm babies born small for gestational age in 138 low-income and middle-income countries in 2010. Lancet Glob. Health.

[154] Li Y., Juhl S.M., Ye X., Shen R.L., Iyore E.O., Dai Y., Sangild P.T., Greisen G.O. (2017). A Stepwise, Pilot Study of Bovine Colostrum to Supplement the First Enteral Feeding in Preterm Infants (Precolos): Study Protocol and Initial Results. Front. Pediatr..

[155] Juhl S.M., Ye X., Zhou P., Li Y., Iyore E.O., Zhang L., Jiang P., van Goudoever J.B., Greisen G., Sangild P.T. (2018). Bovine Colostrum for Preterm Infants in the First Days of Life: A Randomized Controlled Pilot Trial. J. Pediatr. Gastroenterol. Nutr..

[156] Jiang P.P., Muk T., Krych L., Nielsen D.S., Khakimov B., Li Y., Juhl S.M., Greisen G., Sangild P.T. (2021). Gut colonization in preterm infants supplemented with bovine colostrum in the first week of life: An explorative pilot study. J. Parenter. Enter. Nutr..

[157] Hutchens T.W., Henry J.F., Yip T.T. (1991). Structurally intact (78-kDa) forms of maternal lactoferrin purified from urine of preterm infants fed human milk: Identification of a trypsin-like proteolytic cleavage event in vivo that does not result in fragment dissociation. Proc. Natl. Acad. Sci. USA.

[158] Burrin D., Sangild P.T., Stoll B., Thymann T., Buddington R., Marini J., Olutoye O., Shulman R.J. (2020). Translational Advances in Pediatric Nutrition and Gastroenterology: New Insights from Pig Models. Annu. Rev. Anim. Biosci..

[159] Sangild P.T., Thymann T., Schmidt M., Stoll B., Burrin D.G., Buddington R.K. (2013). Invited review: The preterm pig as a model in pediatric gastroenterology. J. Anim. Sci..

[160] Odle J., Lin X., Jacobi S.K., Kim S.W., Stahl C.H. (2014). The suckling piglet as an agrimedical model for the study of pediatric nutrition and metabolism. Annu. Rev. Anim. Biosci..

[161] Sangild P.T., Petersen Y.M., Schmidt M., Elnif J., Petersen T.K., Buddington R.K., Greisen G., Michaelsen K.F., Burrin D.G. (2002). Preterm birth affects the intestinal response to parenteral and enteral nutrition in newborn pigs. J. Nutr..

[162] Bjornvad C.R., Schmidt M., Petersen Y.M., Jensen S.K., Offenberg H., Elnif J., Sangild P.T. (2005). Preterm birth makes the immature intestine sensitive to feeding-induced intestinal atrophy. Am. J. Physiol. Regul. Integr. Comp. Physiol..

[163] Sangild P.T., Siggers R.H., Schmidt M., Elnif J., Bjornvad C.R., Thymann T., Grondahl M.L., Hansen A.K., Jensen S.K., Boye M. (2006). Diet- and colonization-dependent intestinal dysfunction predisposes to necrotizing enterocolitis in preterm pigs. Gastroenterology.

[164] Bjornvad C.R., Thymann T., Deutz N.E., Burrin D.G., Jensen S.K., Jensen B.B., Mølbak L., Boye M., Larsson L.I., Schmidt M. (2008). Enteral feeding induces diet-dependent mucosal dysfunction, bacterial proliferation, and necrotizing enterocolitis in preterm pigs on parenteral nutrition. Am. J. Physiol. Gastrointest. Liver Physiol..

[165] Bæk O., Brunse A., Nguyen D.N., Moodley A., Thymann T., Sangild P.T. (2020). Diet Modulates the High Sensitivity to Systemic Infection in Newborn Preterm Pigs. Front. Immunol..

[166] Brunse A., Worsøe P., Pors S.E., Skovgaard K., Sangild P.T. (2019). Oral Supplementation With Bovine Colostrum Prevents Septic Shock and Brain Barrier Disruption During Bloodstream Infection in Preterm Newborn Pigs. Shock.

[167] Ren S., Hui Y., Obelitz-Ryom K., Brandt A.B., Kot W., Nielsen D.S., Thymann T., Sangild P.T., Nguyen D.N. (2018). Neonatal gut and immune maturation is determined more by postnatal age than by postconceptional age in moderately preterm pigs. Am. J. Physiol. Gastrointest. Liver Physiol..

[168] Hansen C.F., Thymann T., Andersen A.D., Holst J.J., Hartmann B., Hilsted L., Langhorn L., Jelsing J., Sangild P.T. (2016). Rapid gut growth but persistent delay in digestive function in the postnatal period of preterm pigs. Am. J. Physiol. Gastrointest. Liver Physiol..

[169] Andersen A.D., Sangild P.T., Munch S.L., van der Beek E.M., Renes I.B., Ginneken C., Greisen G.O., Thymann T. (2016). Delayed growth, motor function and learning in preterm pigs during early postnatal life. Am. J. Physiol. Regul. Integr. Comp. Physiol..

[170] Bergström A., Kaalund S.S., Skovgaard K., Andersen A.D., Pakkenberg B., Rosenørn A., van Elburg R.M., Thymann T., Greisen G.O., Sangild P.T. (2016). Limited effects of preterm birth and the first enteral nutrition on cerebellum morphology and gene expression in piglets. Physiol. Rep..

[171] Cao M., Andersen A.D., Van Ginneken C., Shen R.L., Petersen S.O., Thymann T., Jing J., Sangild P.T. (2015). Physical activity level is impaired and diet dependent in preterm newborn pigs. Pediatr. Res..

[172] Holme Nielsen C., Bladt Brandt A., Thymann T., Obelitz-Ryom K., Jiang P., Vanden Hole C., van Ginneken C., Pankratova S., Sangild P.T. (2018). Rapid Postnatal Adaptation of Neurodevelopment in Pigs Born Late Preterm. Dev. Neurosci..

[173] Jensen M.L., Sangild P.T., Lykke M., Schmidt M., Boye M., Jensen B.B., Thymann T. (2013). Similar efficacy of human banked milk and bovine colostrum to decrease incidence of necrotizing enterocolitis in preterm piglets. Am. J. Physiol. Regul. Integr. Comp. Physiol..

[174] Rasmussen S.O., Martin L., Østergaard M.V., Rudloff S., Li Y., Roggenbuck M., Bering S.B., Sangild P.T. (2016). Bovine colostrum improves neonatal growth, digestive function, and gut immunity relative to donor human milk and infant formula in preterm pigs. Am. J. Physiol. Gastrointest. Liver Physiol..

[175] Oosterloo B.C., Premkumar M., Stoll B., Olutoye O., Thymann T., Sangild P.T., Burrin D.G. (2014). Dual purpose use of preterm piglets as a model of pediatric GI disease. Vet. Immunol. Immunopathol..

[176] Bæk O., Cilieborg M.S., Bering S.B., Nguyen D.N., Thymann T., Sangild P.T. (2021). Sex-specific survival, growth, immunity and organ development in preterm pigs as models for immature newborns. Front. Pediatr..

[177] Meder U., Tarjanyi E., Kovacs K., Szakmar E., Cseko A.J., Hazay T., Belteki G., Szabo M., Jermendy A. (2020). Cerebral oxygenation in preterm infants during maternal singing combined with skin-to-skin care. Pediatr. Res..

[178] Brunse A., Peng Y., Li Y., Lykkesfeldt J., Sangild P.T. (2021). Co-bedding of preterm newborn pigs reduces necrotizing enterocolitis incidence independent of vital functions and cortisol levels. Front. Pediatr..

[179] Reed R.C., Johnson D.E., Nie A.M. (2021). Preterm Infant Skin Structure Is Qualitatively and Quantitatively Different from That of Term Newborns. Pediatr. Dev. Pathol..

[180] Li J., Xu Y.W., Jiang J.J., Song Q.K. (2019). Bovine colostrum and product intervention associated with relief of childhood infectious diarrhea. Sci. Rep..

[181] Barakat S.H., Meheissen M.A., Omar O.M., Elbana D.A. (2020). Bovine Colostrum in the Treatment of Acute Diarrhea in Children: A Double-Blinded Randomized Controlled Trial. J. Trop. Pediatr..

[182] Premkumar M.H., Massieu L.A., Anderson D.M., Gokulakrishnan G. (2020). Human Milk Supplements: Principles, Practices, and Current Controversies. Clin. Perinatol..

[183] Sun J., Li Y., Nguyen D.N., Mortensen M.S., van den Akker C.H.P., Skeath T., Pors S.E., Pankratova S., Rudloff S., Sørensen S.J. (2018). Nutrient Fortification of Human Donor Milk Affects Intestinal Function and Protein Metabolism in Preterm Pigs. J. Nutr..

[184] Sun J., Li Y., Pan X., Nguyen D.N., Brunse A., Bojesen A.M., Rudloff S., Mortensen M.S., Burrin D.G., Sangild P.T. (2019). Human Milk Fortification with Bovine Colostrum Is Superior to Formula-Based Fortifiers to Prevent Gut Dysfunction, Necrotizing Enterocolitis, and Systemic Infection in Preterm Pigs. J. Parenter. Enter. Nutr..

[185] Boudry C., Buldgen A., Portetelle D., Collard A., Théwis A., Dehoux J.P. (2007). Effects of oral supplementation with bovine colostrum on the immune system of weaned piglets. Res. Vet. Sci..

[186] Boudry C., Dehoux J.P., Wavreille J., Portetelle D., Théwis A., Buldgen A. (2008). Effect of a bovine colostrum whey supplementation on growth performance, faecal Escherichia coli population and systemic immune response of piglets at weaning. Animal.

[187] Poulsen A.R., de Jonge N., Sugiharto S., Nielsen J.L., Lauridsen C., Canibe N. (2017). The microbial community of the gut differs between piglets fed sow milk, milk replacer or bovine colostrum. Br. J. Nutr..

[188] Sugiharto S., Poulsen A.S., Canibe N., Lauridsen C. (2015). Effect of bovine colostrum feeding in comparison with milk replacer and natural feeding on the immune responses and colonisation of enterotoxigenic Escherichia coli in the intestinal tissue of piglets. Br. J. Nutr..

[189] Huguet A., Le Dividich J., Le Huërou-Luron I. (2012). Improvement of growth performance and sanitary status of weaned piglets fed a bovine colostrum-supplemented diet. J. Anim. Sci..

[190] Lallès J.P., Bosi P., Janczyk P., Koopmans S.J., Torrallardona D. (2009). Impact of bioactive substances on the gastrointestinal tract and performance of weaned piglets: A review. Animal.

[191] Hilpert H., Brussow H., Mietens C., Sidoti J., Lerner L., Werchau H. (1987). Use of bovine milk concentrate containing antibody to rotavirus to treat rotavirus gastroenteritis in infants. J. Infect. Dis..

[192] Mitra A.K., Mahalanabis D., Ashraf H., Unicomb L., Eeckels R., Tzipori S. (1995). Hyperimmune cow colostrum reduces diarrhoea due to rotavirus: A double-blind, controlled clinical trial. Acta Paediatr..

[193] Sarker S.A., Casswall T.H., Mahalanabis D., Alam N.H., Albert M.J., Brussow H., Fuchs G.J., Hammerstrom L. (1998). Successful treatment of rotavirus diarrhea in children with immunoglobulin from immunized bovine colostrum. Pediatr. Infect. Dis. J..

[194] Ylitalo S., Uhari M., Rasi S., Pudas J., Leppaluoto J. (1998). Rotaviral antibodies in the treatment of acute rotaviral gastroenteritis. Acta Paediatr..

[195] Mietens C., Keinhorst H., Hilpert H., Gerber H., Amster H., Pahud J.J. (1979). Treatment of infantile E. coli gastroenteritis with specific bovine anti-E. coli milk immunoglobulins. Eur. J. Pediatr..

[196] Casswall T.H., Sarker S.A., Faruque S.M., Weintraub A., Albert M.J., Fuchs G.J., Alam N.H., Dahlstrom A.K., Link H., Brussow H. (2000). Treatment of enterotoxigenic and enteropathogenic Escherichia coli-induced diarrhoea in children with bovine immunoglobulin milk concentrate from hyperimmunized cows: A double-blind, placebo-controlled, clinical trial. Scand. J. Gastroenterol..

[197] Huppertz H.I., Rutkowski S., Busch D.H., Eisebit R., Lissner R., Karch H. (1999). Bovine colostrum ameliorates diarrhea in infection with diarrheagenic Escherichia coli, shiga toxin-producing E. Coli, and E. coli expressing intimin and hemolysin. J. Pediatr. Gastroenterol. Nutr..

[198] Ashraf H., Mahalanabis D., Mitra A.K., Tzipori S., Fuchs G.J. (2001). Hyperimmune bovine colostrum in the treatment of shigellosis in children: A double-blind, randomized, controlled trial. Acta Paediatr..

[199] Ebina T., Sato A., Umezu K., Ishida N., Ohyama S., Ohizumi A., Aikawa K., Katagiri S., Katsushima N., Imai A. (1983). Prevention of rotavirus infection by cow colostrum antibody against human rotaviruses. Lancet.

[200] Davidson G.P., Whyte P.B., Daniels E., Franklin K., Nunan H., McCloud P.I., Moore A.G., Moore D.J. (1989). Passive immunisation of children with bovine colostrum containing antibodies to human rotavirus. Lancet.

[201] Turner R.B., Kelsey D.K. (1993). Passive immunization for prevention of rotavirus illness in healthy infants. Pediatr. Infect. Dis. J..

[202] Brunser O., Espinoza J., Figueroa G., Araya M., Spencer E., Hilpert H., Link-Amster H., Brussow H. (1992). Field trial of an infant formula containing anti-rotavirus and anti-Escherichia coli milk antibodies from hyperimmunized cows. J. Pediatr. Gastroenterol. Nutr..

[203] Den Hartog G., Jacobino S., Bont L., Cox L., Ulfman L.H., Leusen J.H., van Neerven R.J. (2014). Specificity and Effector Functions of Human RSV-Specific IgG from Bovine Milk. PLoS ONE.

[204] Loss G., Depner M., Ulfman L.H., van Neerven R.J., Hose A.J., Genuneit J., Karvonen A.M., Hyvärinen A., Kaulek V., Roduit C. (2015). Consumption of unprocessed cow’s milk protects infants from common respiratory infections. J. Allergy Clin. Immunol..

[205] Von Mutius E., Vercelli D. (2010). Farm living: Effects on childhood asthma and allergy. Nat. Rev. Immunol..

[206] Roselli M., Britti M.S., Le Huërou-Luron I., Marfaing H., Zhu W.Y., Mengheri E. (2007). Effect of different plant extracts and natural substances (PENS) against membrane damage induced by enterotoxigenic Escherichia coli K88 in pig intestinal cells. Toxicol. In Vitro.

[207] De Waard M., Li Y., Zhu Y., Ayede A.I., Berrington J., Bloomfield F.H., Busari O.O., Cormack B.E., Embleton N.D., van Goudoever J.B. (2019). Time to Full Enteral Feeding for Very Low-Birth-Weight Infants Varies Markedly Among Hospitals Worldwide But May Not Be Associated With Incidence of Necrotizing Enterocolitis: The NEOMUNE-NeoNutriNet Cohort Study. J. Parenter. Enter. Nutr..

[208] Sadeghirad B., Morgan R.L., Zeraatkar D., Zea A.M., Couban R., Johnston B.C., Florez I.D. (2018). Human and Bovine Colostrum for Prevention of Necrotizing Enterocolitis: A Meta-analysis. Pediatrics.

[209] Ahnfeldt A.M., Hyldig N., Li Y., Kappel S.S., Aunsholdt L., Sangild P.T., Zachariassen G. (2019). FortiColos—A multicentre study using bovine colostrum as a fortifier to human milk in very preterm infants: Study protocol for a randomised controlled pilot trial. Trials.

[210] Brooks H.J., McConnell M.A., Corbett J., Buchan G.S., Fitzpatrick C.E., Broadbent R.S. (2006). Potential prophylactic value of bovine colostrum in necrotizing enterocolitis in neonates: An in vitro study on bacterial attachment, antibody levels and cytokine production. FEMS Immunol. Med. Microbiol..

[211] Balachandran B., Dutta S., Singh R., Prasad R., Kumar P. (2017). Bovine Colostrum in Prevention of Necrotizing Enterocolitis and Sepsis in Very Low Birth Weight Neonates: A Randomized, Double-blind, Placebo-controlled Pilot Trial. J. Trop. Pediatr..

[212] Ismail R.I.H., Awad H.A., Imam S.S., Gad G.I., Aboushady N.M., Abdou R.M., Eissa D.S., Azzam N.T., Barakat M.M., Yassin M.M. (2021). Gut priming with bovine colostrum and T regulatory cells in preterm neonates: A randomized controlled trial. Pediatr. Res..

[213] Eibl M.M., Wolf H.M., Furnkranz H., Rosenkranz A. (1988). Prevention of necrotizing enterocolitis in low-birth-weight infants by IgA-IgG feeding. N. Engl. J. Med..

[214] Lawrence G., Tudehope D., Baumann K., Jeffery H., Gill A., Cole M., Drew J., McPhee A., Ratcliffe J., Reynolds G. (2001). Enteral human IgG for prevention of necrotising enterocolitis: A placebo-controlled, randomised trial. Lancet.

[215] Rubaltelli F.F., Benini F., Sala M. (1991). Prevention of necrotizing enterocolitis in neonates at risk by oral administration of monomeric IgG. Dev. Pharmacol. Ther..

[216] Fast C., Rosegger H. (1994). Necrotizing enterocolitis prophylaxis: Oral antibiotics and lyophilized enterobacteria vs. oral immunoglobulins. Acta Paediatr..

[217] Richter D., Bartmann P., Pohlandt F. (1998). Prevention of necrotizing enterocolitis in extremely low birth weight infants by IgG feeding?. Eur. J. Pediatr..

[218] Foster J.P., Seth R., Cole M.J. (2016). Oral immunoglobulin for preventing necrotizing enterocolitis in preterm and low birth weight neonates. Cochrane Database Syst. Rev..

[219] Siggers J., Sangild P.T., Jensen T.K., Siggers R.H., Skovgaard K., Støy A.C., Jensen B.B., Thymann T., Bering S.B., Boye M. (2011). Transition from parenteral to enteral nutrition induces immediate diet-dependent gut histological and immunological responses in preterm neonates. Am. J. Physiol. Gastrointest. Liver Physiol..

[220] Danielsen M., Thymann T., Jensen B.B., Jensen O.N., Sangild P.T., Bendixen E. (2006). Proteome profiles of mucosal immunoglobulin uptake in inflamed porcine gut. Proteomics.

[221] Che L., Thymann T., Bering S.B., Le Huerou-Luron I., D’inca R., Zhang K., Sangild P.T. (2010). IUGR does not predispose to necrotizing enterocolitis or compromise postnatal intestinal adaptation in preterm pigs. Pediatr. Res..

[222] Cilieborg M.S., Boye M., Mølbak L., Thymann T., Sangild P.T. (2011). Preterm birth and necrotizing enterocolitis alter gut colonization in pigs. Pediatr. Res..

[223] Cilieborg M.S., Boye M., Thymann T., Jensen B.B., Sangild P.T. (2011). Diet-dependent effects of minimal enteral nutrition on intestinal function and necrotizing enterocolitis in preterm pigs. J. Parenter. Enter. Nutr..

[224] Li Y., Jensen M.L., Chatterton D.E., Jensen B.B., Thymann T., Kvistgaard A.S., Sangild P.T. (2014). Raw bovine milk improves gut responses to feeding relative to infant formula in preterm piglets. Am. J. Physiol. Gastrointest. Liver Physiol..

[225] Li Y., Østergaard M.V., Jiang P., Chatterton D.E., Thymann T., Kvistgaard A.S., Sangild P.T. (2013). Whey protein processing influences formula-induced gut maturation in preterm pigs. J. Nutr..

[226] Møller H.K., Thymann T., Fink L.N., Frokiaer H., Kvistgaard A.S., Sangild P.T. (2011). Bovine colostrum is superior to enriched formulas in stimulating intestinal function and necrotising enterocolitis resistance in preterm pigs. Br. J. Nutr..

[227] Oste M., Van Ginneken C.J., Van Haver E.R., Bjornvad C.R., Thymann T., Sangild P.T. (2005). The intestinal trophic response to enteral food is reduced in parenterally fed preterm pigs and is associated with more nitrergic neurons. J. Nutr..

[228] Oste M., Van Haver E., Thymann T., Sangild P., Weyns A., Van Ginneken C.J. (2010). Formula induces intestinal apoptosis in preterm pigs within a few hours of feeding. J. Parenter. Enter. Nutr..

[229] Shen R.L., Thymann T., Østergaard M.V., Støy A.C., Krych Ł., Nielsen D.S., Lauridsen C., Hartmann B., Holst J.J., Burrin D.G. (2015). Early gradual feeding with bovine colostrum improves gut function and NEC resistance relative to infant formula in preterm pigs. Am. J. Physiol. Gastrointest. Liver Physiol..

[230] Siggers J., Ostergaard M.V., Siggers R.H., Skovgaard K., Mølbak L., Thymann T., Schmidt M., Møller H.K., Purup S., Fink L.N. (2013). Postnatal amniotic fluid intake reduces gut inflammatory responses and necrotizing enterocolitis in preterm neonates. Am. J. Physiol. Gastrointest. Liver Physiol..

[231] Siggers R.H., Thymann T., Jensen B.B., Mølbak L., Heegaard P.M., Schmidt M., Buddington R.K., Sangild P.T. (2008). Elective cesarean delivery affects gut maturation and delays microbial colonization but does not increase necrotizing enterocolitis in preterm pigs. Am. J. Physiol. Regul. Integr. Comp. Physiol..

[232] Sty A.C., Sangild P.T., Skovgaard K., Thymann T., Bjerre M., Chatterton D.E., Purup S., Boye M., Heegaard P.M. (2016). Spray Dried, Pasteurised Bovine Colostrum Protects Against Gut Dysfunction and Inflammation in Preterm Pigs. J. Pediatr. Gastroenterol. Nutr..

[233] Støy A.C., Heegaard P.M., Thymann T., Bjerre M., Skovgaard K., Boye M., Stoll B., Schmidt M., Jensen B.B., Sangild P.T. (2014). Bovine colostrum improves intestinal function following formula-induced gut inflammation in preterm pigs. Clin. Nutr..

[234] Van Haver E.R., de Vooght L., Oste M., Sangild P.T., Thymann T., Weyns A.L., van Ginneken C.J. (2008). Postnatal and diet-dependent increases in enteric glial cells and VIP-containing neurones in preterm pigs. Neurogastroenterol. Motil..

[235] Van Haver E.R., Oste M., Thymann T., Sys S.U., Lamers W.H., Weyns A.L., Sangild P.T., van Ginneken C.J. (2008). Enteral feeding reduces endothelial nitric oxide synthase in the caudal intestinal microvasculature of preterm piglets. Pediatr. Res..

[236] Van Haver E.R., Sangild P.T., Oste M., Siggers J.L., Weyns A.L., Van Ginneken C.J. (2009). Diet-dependent mucosal colonization and interleukin-1beta responses in preterm pigs susceptible to necrotizing enterocolitis. J. Pediatr. Gastroenterol. Nutr..

[237] Willems R., Krych L., Rybicki V., Jiang P., Sangild P.T., Shen R.L., Hensel K.O., Wirth S., Postberg J., Jenke A.C. (2015). Introducing enteral feeding induces intestinal subclinical inflammation and respective chromatin changes in preterm pigs. Epigenomics.

[238] Ren S., Hui Y., Goericke-Pesch S., Pankratova S., Kot W., Pan X., Thymann T., Sangild P.T., Nguyen D.N. (2019). Gut and immune effects of bioactive milk factors in preterm pigs exposed to prenatal inflammation. Am. J. Physiol. Gastrointest. Liver Physiol..

[239] Li Y., Pan X., Nguyen D.N., Ren S., Moodley A., Sangild P.T. (2019). Bovine Colostrum Before or After Formula Feeding Improves Systemic Immune Protection and Gut Function in Newborn Preterm Pigs. Front. Immunol..

[240] Yan X., Sangild P.T., Peng Y., Li Y., Bering S.B., Pan X. (2021). Supplementary bovine colostrum feeding to formula-fed preterm pigs improves gut function and reduces necrotizing enterocolitis. J. Pediatr. Gastroenterol. Nutr..

[241] Buddington R.K., Bering S.B., Thymann T., Sangild P.T. (2008). Aldohexose malabsorption in preterm pigs is directly related to the severity of necrotizing enterocolitis. Pediatr. Res..

[242] Oste M., De Vos M., Van Haver E., Van Brantegem L., Thymann T., Sangild P., Weyns A., Van Ginneken C. (2010). Parenteral and enteral feeding in preterm piglets differently affects extracellular matrix proteins, enterocyte proliferation and apoptosis in the small intestine. Br. J. Nutr..

[243] Puiman P.J., Jensen M., Stoll B., Renes I.B., de Bruijn A.C., Dorst K., Schierbeek H., Schmidt M., Boehm G., Burrin D.G. (2011). Intestinal threonine utilization for protein and mucin synthesis is decreased in formula-fed preterm pigs. J. Nutr..

[244] Cilieborg M.S., Boye M., Sangild P.T. (2012). Bacterial colonization and gut development in preterm neonates. Early Hum. Dev..

[245] Wolfs T.G., Jellema R.K., Turrisi G., Becucci E., Buonocore G., Kramer B.W. (2012). Inflammation-induced immune suppression of the fetus: A potential link between chorioamnionitis and postnatal early onset sepsis. J. Matern. Fetal Neonatal Med..

[246] Ophelders D., Gussenhoven R., Klein L., Jellema R.K., Westerlaken R.J.J., Hütten M.C., Vermeulen J., Wassink G., Gunn A.J., Wolfs T. (2020). Preterm Brain Injury, Antenatal Triggers, and Therapeutics: Timing Is Key. Cells.

[247] Gussenhoven R., Westerlaken R.J.J., Ophelders D., Jobe A.H., Kemp M.W., Kallapur S.G., Zimmermann L.J., Sangild P.T., Pankratova S., Gressens P. (2018). Chorioamnionitis, neuroinflammation, and injury: Timing is key in the preterm ovine fetus. J. Neuroinflamm..

[248] Cilieborg M.S., Schmidt M., Skovgaard K., Boye M., Weber N.R., Heegaard P.M., Burrin D.G., Sangild P.T. (2011). Fetal lipopolysaccharide exposure modulates diet-dependent gut maturation and sensitivity to necrotising enterocolitis in pre-term pigs. Br. J. Nutr..

[249] Coggins S.A., Laskin B., Harris M.C., Grundmeier R.W., Passarella M., McKenna K.J., Srinivasan L. (2021). Acute Kidney Injury Associated with Late-Onset Neonatal Sepsis: A Matched Cohort Study. J. Pediatr..

[250] Gao X., Li Y., Olin A.B., Nguyen D.N. (2020). Fortification with bovine colostrum enhances antibacterial activity of human milk. J. Parenter. Enter. Nutr..

[251] Harbeson D., Francis F., Bao W., Amenyogbe N.A., Kollmann T.R. (2018). Energy Demands of Early Life Drive a Disease Tolerant Phenotype and Dictate Outcome in Neonatal Bacterial Sepsis. Front. Immunol..

[252] Trend S., Strunk T., Hibbert J., Kok C.H., Zhang G., Doherty D.A., Richmond P., Burgner D., Simmer K., Davidson D.J. (2015). Antimicrobial protein and Peptide concentrations and activity in human breast milk consumed by preterm infants at risk of late-onset neonatal sepsis. PLoS ONE.

[253] Li Y., Nguyen D.N., de Waard M., Christensen L., Zhou P., Jiang P., Sun J., Bojesen A.M., Lauridsen C., Lykkesfeldt J. (2017). Pasteurization Procedures for Donor Human Milk Affect Body Growth, Intestinal Structure, and Resistance against Bacterial Infections in Preterm Pigs. J. Nutr..

[254] Nguyen D.N., Currie A., Ren S., Bering S., Sangild P. (2019). Heat treatment and irradiation reduce anti-bacterial and immune-modulatory properties of bovine colostrum. J. Funct. Foods.

[255] Playford R.J., Garbowsky M., Marchbank T. (2020). Pasteurized Chicken Egg Powder Stimulates Proliferation and Migration of AGS, RIE1, and Caco-2 Cells and Reduces NSAID-Induced Injury in Mice and Colitis in Rats. J. Nutr..

[256] Ye Y., Manne S., Treem W.R., Bennett D. (2020). Prevalence of Inflammatory Bowel Disease in Pediatric and Adult Populations: Recent Estimates from Large National Databases in the United States, 2007–2016. Inflamm. Bowel Dis..

[257] Cucinotta U., Romano C., Dipasquale V. (2021). Diet and Nutrition in Pediatric Inflammatory Bowel Diseases. Nutrients.

[258] Khan Z., Macdonald C., Wicks A.C., Holt M.P., Floyd D., Ghosh S., Wright N.A., Playford R.J. (2002). Use of the ‘nutriceutical’, bovine colostrum, for the treatment of distal colitis: Results from an initial study. Aliment. Pharmacol. Ther..

[259] Bodammer P., Kerkhoff C., Maletzki C., Lamprecht G. (2013). Bovine colostrum increases pore-forming claudin-2 protein expression but paradoxically not ion permeability possibly by a change of the intestinal cytokine milieu. PLoS ONE.

[260] Filipescu I.E., Leonardi L., Menchetti L., Guelfi G., Traina G., Casagrande-Proietti P., Piro F., Quattrone A., Barbato O., Brecchia G. (2018). Preventive effects of bovine colostrum supplementation in TNBS-induced colitis in mice. PLoS ONE.

[261] Sangild P.T., Ney D.M., Sigalet D.L., Vegge A., Burrin D. (2014). Animal models of gastrointestinal and liver diseases. Animal models of infant short bowel syndrome: Translational relevance and challenges. Am. J. Physiol. Gastrointest. Liver Physiol..

[262] Aunsholt L., Jeppesen P.B., Lund P., Sangild P.T., Ifaoui I.B., Qvist N., Husby S. (2014). Bovine colostrum to children with short bowel syndrome: A randomized, double-blind, crossover pilot study. J. Parenter. Enter. Nutr..

[263] Aunsholt L., Qvist N., Sangild P.T., Vegge A., Stoll B., Burrin D.G., Jeppesen P.B., Eriksen T., Husby S., Thymann T. (2018). Minimal Enteral Nutrition to Improve Adaptation After Intestinal Resection in Piglets and Infants. J. Parenter. Enter. Nutr..

[264] Aunsholt L., Thymann T., Qvist N., Sigalet D., Husby S., Sangild P.T. (2015). Prematurity Reduces Functional Adaptation to Intestinal Resection in Piglets. J. Parenter. Enter. Nutr..

[265] Vegge A., Thymann T., Lund P., Stoll B., Bering S.B., Hartmann B., Jelsing J., Qvist N., Burrin D.G., Jeppesen P.B. (2013). Glucagon-like peptide-2 induces rapid digestive adaptation following intestinal resection in preterm neonates. Am. J. Physiol. Gastrointest. Liver Physiol..

[266] Nagy E.S., Paris M.C., Taylor R.G., Fuller P.J., Sourial M., Justice F., Bines J.E. (2004). Colostrum protein concentrate enhances intestinal adaptation after massive small bowel resection in juvenile pigs. J. Pediatr. Gastroenterol. Nutr..

[267] Pereira-Fantini P.M., Thomas S.L., Taylor R.G., Nagy E., Sourial M., Fuller P.J., Bines J.E. (2008). Colostrum supplementation restores insulin-like growth factor -1 levels and alters muscle morphology following massive small bowel resection. J. Parenter. Enter. Nutr..

[268] Paris M.C., Fuller P.J., Carstensen B., Nagy E., Taylor R.G., Sourial M., Holst J.J., Hartmann B., Binesm J.E. (2004). Plasma GLP-2 levels and intestinal markers in the juvenile pig during intestinal adaptation: Effects of different diet regimens. Dig. Dis. Sci..

[269] Sangild P.T., Shen R.L., Pontoppidan P., Rathe M. (2018). Animal models of chemotherapy-induced mucositis: Translational relevance and challenges. Am. J. Physiol. Gastrointest. Liver Physiol..

[270] Rathe M., De Pietri S., Wehner P.S., Frandsen T.L., Grell K., Schmiegelow K., Sangild P.T., Husby S., Muller K. (2020). Bovine Colostrum Against Chemotherapy-Induced Gastrointestinal Toxicity in Children With Acute Lymphoblastic Leukemia: A Randomized, Double-Blind, Placebo-Controlled Trial. J. Parenter. Enter. Nutr..

[271] Martin J., Howard S.C., Pillai A., Vogel P., Naren A.P., Davis S., Ringwald-Smith K., Buddington K., Buddington R.K. (2014). The weaned pig as a model for Doxorubicin-induced mucositis. Chemotherapy.

[272] Shen R.L., Pontoppidan P.E., Rathe M., Jiang P., Hansen C.F., Buddington R.K., Heegaard P.M., Müller K., Sangild P.T. (2016). Milk diets influence doxorubicin-induced intestinal toxicity in piglets. Am. J. Physiol. Gastrointest. Liver Physiol..

[273] Shen R.L., Rathe M., Jiang P., Pontoppidan P.E., Heegaard P.M., Müller K., Sangild P.T. (2016). Doxorubicin-Induced Gut Toxicity in Piglets Fed Bovine Milk and Colostrum. J. Pediatr. Gastroenterol. Nutr..

[274] Pontoppidan P.E., Shen R.L., Cilieborg M.S., Jiang P., Kissow H., Petersen B.L., Thymann T., Heilmann C., Muller K., Sangild P.T. (2015). Bovine Colostrum Modulates Myeloablative Chemotherapy-Induced Gut Toxicity in Piglets. J. Nutr..

[275] Pontoppidan P.L., Shen R.L., Petersen B.L., Thymann T., Heilmann C., Müller K., Sangild P.T. (2014). Intestinal response to myeloablative chemotherapy in piglets. Exp. Biol. Med..

[276] Chatterton D.E.W., Aagaard S., Hesselballe Hansen T., Nguyen D.N., De Gobba C., Lametsch R., Sangild P.T. (2020). Bioactive proteins in bovine colostrum and effects of heating, drying and irradiation. Food Funct..

[277] Elfstrand L., Lindmark-Månsson H., Paulsson M., Nyberg L., Åkesson B. (2002). Immunoglobulins, growth factors and growth hormone in bovine colostrum and the effects of processing. Int. Dairy J..

[278] Foster D.M., Poulsen K.P., Sylvester H.J., Jacob M.E., Casulli K.E., Farkas B.E. (2016). Effect of high-pressure processing of bovine colostrum on immunoglobulin G concentration, pathogens, viscosity, and transfer of passive immunity to calves. J. Dairy Sci..

[279] Navis M., Muncan V., Sangild P.T., Møller Willumsen L., Koelink P.J., Wildenberg M.E., Abrahamse E., Thymann T., van Elburg R.M., Renes I.B. (2020). Beneficial Effect of Mildly Pasteurized Whey Protein on Intestinal Integrity and Innate Defense in Preterm and Near-Term Piglets. Nutrients.

[280] Navis M., Schwebel L., Soendergaard Kappel S., Muncan V., Sangild P.T., Abrahamse E., Aunsholt L., Thymann T., van Elburg R.M., Renes I.B. (2020). Mildly Pasteurized Whey Protein Promotes Gut Tolerance in Immature Piglets Compared with Extensively Heated Whey Protein. Nutrients.

[281] Lönnerdal B., Hernell O. (1998). Effects of feeding ultrahigh-temperature (UHT)-treated infant formula with different protein concentrations or powdered formula, as compared with breast-feeding, on plasma amino acids, hematology, and trace element status. Am. J. Clin. Nutr..

[282] Playford R.J., Cattell M., Marchbank T. (2020). Marked variability in bioactivity between commercially available bovine colostrum for human use; implications for clinical trials. PLoS ONE.

[283] Klobasa F., Butler J.E., Habe F. (1990). Maternal-neonatal immunoregulation: Suppression of de novo synthesis of IgG and IgA, but not IgM, in neonatal pigs by bovine colostrum, is lost upon storage. Am. J. Vet. Res..

[284] Agbokounou A.M., Ahounou G.S., Youssao Abdou Karim I., Mensah G.A., Koutinhouin B., Hornick J.L. (2017). Effect of cow colostrum on the performance and survival rate of local newborn piglets in Benin Republic. Trop. Anim. Health Prod..

[285] Liu Y., Zhang W., Han B., Zhang L., Zhou P. (2020). Changes in bioactive milk serum proteins during milk powder processing. Food Chem..

[286] Langhendries J.P., Hurrell R.F., Furniss D.E., Hischenhuber C., Finot P.A., Bernard A., Battisti O., Bertrand J.M., Senterre J. (1992). Maillard reaction products and lysinoalanine: Urinary excretion and the effects on kidney function of preterm infants fed heat-processed milk formula. J. Pediatr. Gastroenterol. Nutr..

[287] Cattaneo S., Masotti F., Pellegrino L. (2009). Liquid infant formulas: Technological tools for limiting heat damage. J. Agric. Food Chem..

[288] Cano-Sancho G., Alexandre-Gouabau M.C., Moyon T., Royer A.L., Guitton Y., Billard H., Darmaun D., Rozé J.C., Boquien C.Y., Le Bizec B. (2020). Simultaneous exploration of nutrients and pollutants in human milk and their impact on preterm infant growth: An integrative cross-platform approach. Environ. Res..

[289] Andrew S.M. (2001). Effect of composition of colostrum and transition milk from Holstein heifers on specificity rates of antibiotic residue tests. J. Dairy Sci..

[290] an Wattum J.J., Leferink T.M., Wilffert B., Ter Horst P.G.J. (2019). Antibiotics and lactation: An overview of relative infant doses and a systematic assessment of clinical studies. Basic Clin. Pharmacol. Toxicol..

[291] Hair A.B., Blanco C.L., Moreira A.G., Hawthorne K.M., Lee M.L., Rechtman D.J., Abrams S.A. (2014). Randomized trial of human milk cream as a supplement to standard fortification of an exclusive human milk-based diet in infants 750-1250 g birth weight. J. Pediatr..

[292] Rechtman D.J., Lee M.L., Berg H. (2006). Effect of environmental conditions on unpasteurized donor human milk. Breastfeed. Med. Off. J. Acad. Breastfeed. Med..

[293] Salcedo J., Karav S., Le Parc A., Cohen J.L., de Moura Bell J., Sun A., Lange M.C., Barile D. (2018). Applihairations of industrial treatments to donor human milk: Influence of pasteurization treatments, storage temperature, and time on human milk gangliosides. NPJ Sci. Food.

[294] Høst A. (1994). Cow’s milk protein allergy and intolerance in infancy. Some clinical, epidemiological and immunological aspects. Pediatr. Allergy Immunol..

[295] Barni S., Giovannini M., Mori F. (2021). Epidemiology of non-IgE-mediated food allergies: What can we learn from that?. Curr. Opin. Allergy Clin. Immunol..

[296] Cianferoni A. (2021). Food protein-induced enterocolitis syndrome epidemiology. Ann. Allergy Asthma Immunol..

[297] Matsumoto N., Okochi M., Matsushima M., Kato R., Takase T., Yoshida Y., Kawase M., Isobe K., Kawabe T., Honda H. (2009). Peptide array-based analysis of the specific IgE and IgG4 in cow’s milk allergens and its use in allergy evaluation. Peptides.

[298] Villa C., Costa J., Oliveira M., Mafra I. (2018). Bovine Milk Allergens: A Comprehensive Review. Compr. Rev. Food Sci. Food Saf..

[299] Zachariassen G., Faerk J., Esberg B.H., Fenger-Gron J., Mortensen S., Christesen H.T., Halken S. (2011). Allergic diseases among very preterm infants according to nutrition after hospital discharge. Pediatr. Allergy Immunol..

[300] Thureen P.J., Melara D., Fennessey P.V., Hay W.W. (2003). Effect of low versus high intravenous amino acid intake on very low birth weight infants in the early neonatal period. Pediatr. Res..

[301] Hay W.W. (2008). Strategies for feeding the preterm infant. Neonatology.

[302] Clark D., Henderson M., Smith M., Dear P.R. (1989). Plasma amino acid concentrations in parenterally fed preterm infants. Arch. Dis. Child..

[303] Meyer R., Foong R.X., Thapar N., Kritas S., Shah N. (2015). Systematic review of the impact of feed protein type and degree of hydrolysis on gastric emptying in children. BMC Gastroenterol..

[304] Ghoneim N., Bauchart-Thevret C., Oosterloo B., Stoll B., Kulkarni M., de Pipaon M.S., Zamora I.J., Olutoye O.O., Berg B., Wittke A. (2014). Delayed initiation but not gradual advancement of enteral formula feeding reduces the incidence of necrotizing enterocolitis (NEC) in preterm pigs. PLoS ONE.

[305] Corvaglia L., Mariani E., Aceti A., Galletti S., Faldella G. (2013). Extensively hydrolyzed protein formula reduces acid gastro-esophageal reflux in symptomatic preterm infants. Early Hum. Dev..

[306] Ferreira C.H.F., Martinez F.E., Crott G.C., Belik J. (2018). Gavage Feed Volume Determines the Gastric Emptying Rate in Preterm Infants. J. Pediatr. Gastroenterol. Nutr..

[307] Perrella S.L., Hepworth A.R., Gridneva Z., Simmer K.N., Hartmann P.E., Geddes D.T. (2015). Gastric Emptying and Curding of Pasteurized Donor Human Milk and Mother’s Own Milk in Preterm Infants. J. Pediatr. Gastroenterol. Nutr..

[308] Thymann T., Støy C.A., Bering S.B., Mølbak L., Sangild P.T. (2012). Casein addition to a whey-based formula has limited effects on gut function in preterm pigs. J. Anim. Sci..

[309] Kappel S.S., Sangild P.T., Scheike T., Friborg C.R., Gormsen M., Aunsholt L. (2020). Radiographic Imaging to Evaluate Food Passage Rate in Preterm Piglets as a Model for Preterm Infants. Front. Pediatr..

[310] Buddington R.K., Davis S.L., Buddington K.K. (2018). The Risk of Necrotizing Enterocolitis Differs Among Preterm Pigs Fed Formulas With Either Lactose or Maltodextrin. J. Pediatr. Gastroenterol. Nutr..

